# Stachybotrychromenes A–C: novel cytotoxic meroterpenoids from *Stachybotrys* sp.

**DOI:** 10.1007/s12550-018-0312-7

**Published:** 2018-03-16

**Authors:** Annika Jagels, Yannick Hövelmann, Alexa Zielinski, Melanie Esselen, Jens Köhler, Florian Hübner, Hans-Ulrich Humpf

**Affiliations:** 1 Institute of Food Chemistry, Westfälische Wilhelms-Universität Münster, Corrensstr. 45, 48149 Münster, Germany; 2 Institute of Pharmaceutical and Medicinal Chemistry, Westfälische Wilhelms-Universität Münster, Corrensstr. 48, 48149 Münster, Germany

**Keywords:** Mycotoxins, *Stachybotrys*, Meroterpenoids, Isolation, Structure elucidation, Cytotoxicity

## Abstract

In the course of gaining new insights into the secondary metabolite profile of various *Stachybotrys* strains, in particular concerning triprenyl phenol-like compounds, so far, unknown metabolites with analogous structural features were discovered. Three novel meroterpenoids containing a chromene ring moiety, namely stachybotrychromenes A–C, were isolated from solid culture of the filamentous fungus *Stachybotrys chartarum* DSMZ 12880 (chemotype S). Their structures were elucidated by means of comprehensive spectroscopic analysis (1D and 2D NMR, ESI-HRMS, and CD) as well as by comparison with spectroscopic data of structural analogues described in literature. Stachybotrychromenes A and B exhibited moderate cytotoxic effects on HepG2 cells after 24 h with corresponding IC_50_ values of 73.7 and 28.2 μM, respectively. Stachybotrychromene C showed no significant cytotoxic activity up to 100 μM. Moreover, it is noteworthy that stachybotrychromenes A–C are produced not only by *S. chartarum* chemotype S but also *S. chartarum* chemotype A and *Stachybotrys chlorohalonata*.

**Electronic supplementary material:**

The online version of this article (10.1007/s12550-018-0312-7) contains supplementary material, which is available to authorized users.

## Introduction

**Fig. 1:**
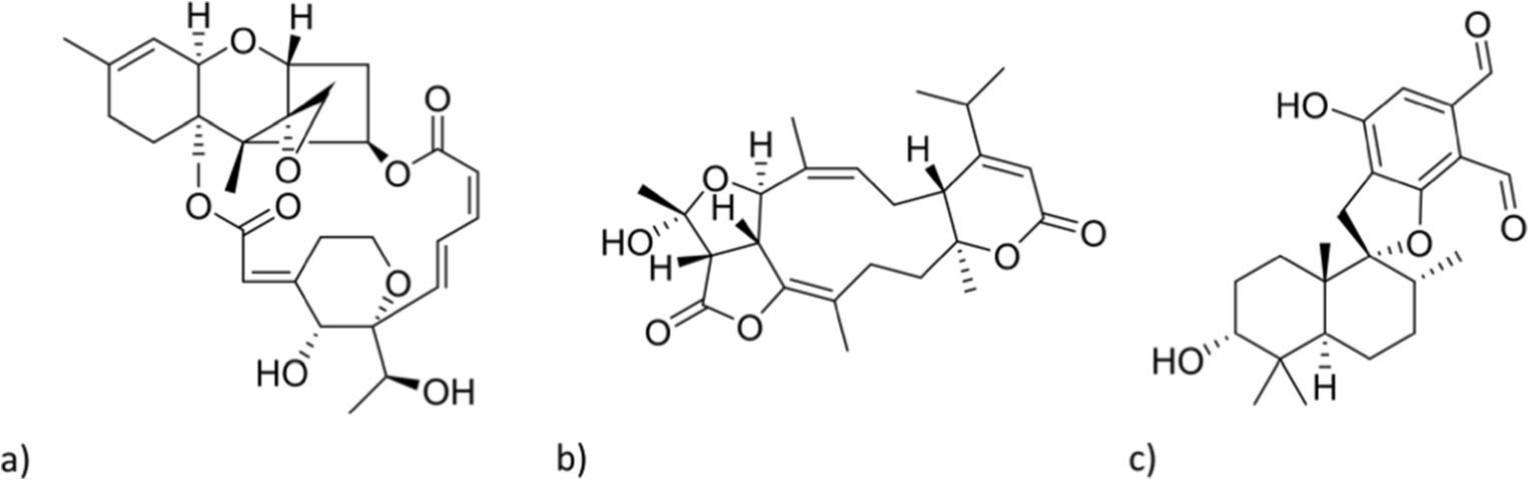
Main classes of *Stachybotrys* metabolites. **a** Macrocyclic trichothecenes (satratoxin H). **b** Atranones (atranone A). **c** Phenylspirodrimanes (stachybotrydial)

The fungal genus *Stachybotrys* is ubiquitously present in the environment, especially on commodities rich in cellulose, such as straw, wood, and paper as well as gypsum board, and was first isolated from a moldy wallpaper in Prague (Bisby 1943). The occurrence of *Stachybotrys* species has been reported frequently, particularly in water-damaged buildings (Johanning et al. 1996; Jarvis et al. 2000; Bloom et al. 2009). In this context, the species *Stachybotry*s *chartarum* (*atra*) received attention in the 1990s, within cases of building-related illnesses and idiopathic pulmonary hemosiderosis in infants (Etzel et al. 1998; Dearborn et al. 1999; Page and Trout 2001; Pestka et al. 2008). Approximately 150 secondary metabolites from *Stachybotrys* species are known and might be related to the observed adverse human health effects in the cases mentioned above (Jarvis et al. 1998; Hossain et al. 2004). Important representatives are macrocyclic trichothecenes, including the highly toxic satratoxins, atranones, and phenylspirodrimanes (Fig. 1) (Jarvis 2003). The phenylspirodrimanes occur in considerably higher levels and are therefore designated to be the most characteristic and dominant group of mycotoxins among this genus (Jarvis et al. 1995). Additionally, the latter compounds are produced by the majority of *Stachybotrys* species, whereas the macrocyclic trichothecenes are only produced by *S. chartarum* chemotype S (Andersen et al. 2003).

Phenylspirodrimanes are part of the group of triprenyl phenols, which in turn belong to the broad group of meroterpenoids. The latter represent natural products whose molecular structures contain moieties originating from the polyketide pathway as well the terpenoid pathway (Geris and Simpson 2009; Matsuda and Abe 2016). This structurally unique class of substances shows a broad diversity of biological activities. For instance, disruption of the complement system, immunotoxicity, neurotoxicity, cytotoxicity, fibrinolysis, and plasminogen activation, as well as antiviral and antiplasmodial activity, have been shown (Sawadjoon et al. 2004; Hasumi et al. 2007; Sasaoka et al. 2007; Wang et al. 2015).

Particularly in the last years, the genus *Stachybotrys* revealed tremendous potential for the isolation of novel meroterpenoid derivatives with remarkable properties concerning biological activity, e.g., inhibitory effects against the dengue virus (Li et al. 2014a, b; Bao et al. 2015; Ma et al. 2015; Chunyu et al. 2016; Ding et al. 2017a, b; Li et al. 2017; Liu et al. 2017; Yin et al. 2017, Zhang et al. 2017; Zhao et al. 2017a, b, c).

**Fig. 2:**
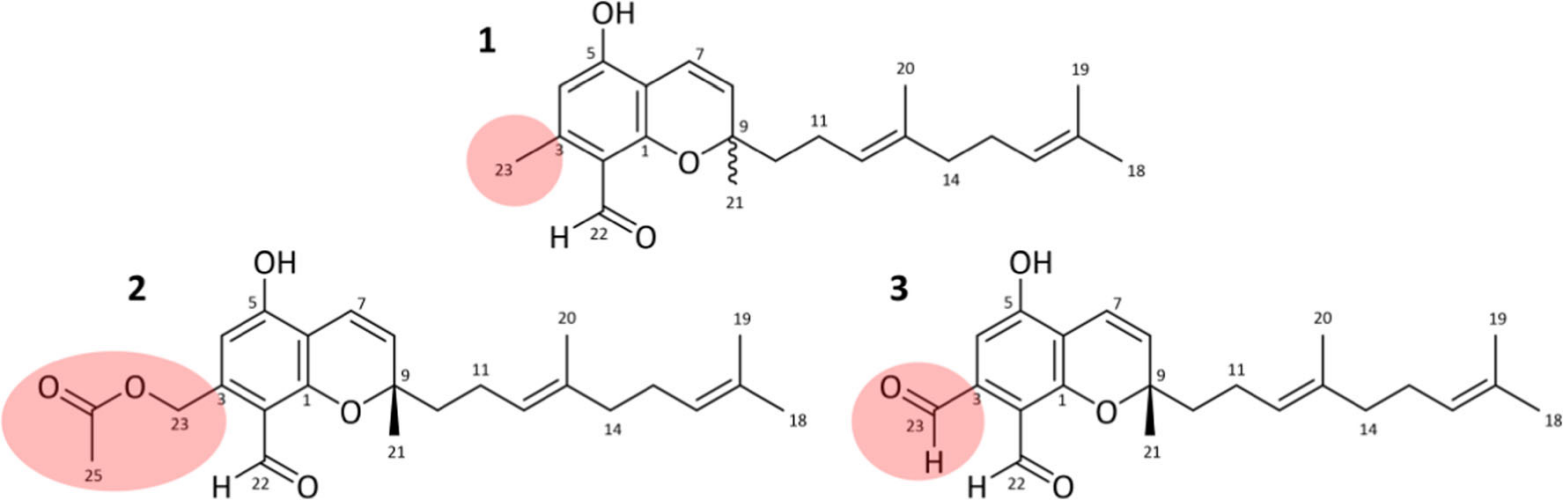
Chemical structures of the isolated stachybotrychromenes A–C (**1**–**3**) from *S. chartarum*

In the course of HPLC-MS/MS profiling of secondary metabolites from *Stachybotrys* species, the triprenyl phenol-like compounds stachybotrychromenes A–C (**1**–**3**) (Fig. 2) were identified as three novel meroterpenoid metabolites. Herein, we describe the isolation and structure elucidation and give first insights into cytotoxicity of these compounds.

## Materials and methods

### General experimental procedures

Analytical thin-layer chromatography (TLC) was performed with pre-coated silica gel plates (Silica gel 60 F254, Merck, Darmstadt, Germany), and UV irradiation was used for visualization (254 and 360 nm, Herolab, Wiesloch, Germany).

Semi-preparative reversed-phase high-performance liquid chromatography with ultraviolet detection (RP-HPLC-UV) was carried out by using a Jasco system (Jasco, Groß-Umstadt, Germany) equipped with a 1-mL sample loop (Rheodyne, CA, USA) at a wavelength of 360 nm with an Agilent Eclipse XDB-C 18 column (250 × 9.4 mm, 5 μm, Agilent, Waldbronn, Germany).

1D and 2D nuclear magnetic resonance (NMR) spectra were recorded at 600 MHz for ^1^H NMR and 150 MHz for ^13^C NMR, respectively, using an Agilent DD2 600 MHz spectrometer (Agilent, Waldbronn, Germany). Chemical shifts are reported in parts per million (ppm), coupling constants (*J*) are given in Hertz (Hz), and CD_3_CN was used as the solvent.

High-resolution mass spectrometry with electrospray ionization (ESI-HRMS) data were obtained using an LTQ Orbitrap XL instrument (Thermo Fisher Scientific, Bremen, Germany). UV spectra were obtained with a J-750 Spectrophotometer (Jasco, Groß-Umstadt, Germany) in acetonitrile (MeCN).

For circular dichroism (CD) measurements, a J-600 Spectropolarimeter (Jasco, Groß-Umstadt, Germany) with a 0.1-cm cell in MeCN at room temperature was used.

### Chemicals and culture media

Ethyl acetate (EtOAc, analytical grade) was purchased from Grüssing (Filsum, Germany). Cyclohexane (synthesis grade) and MeCN (HPLC grade) were purchased from Roth (Karlsruhe, Germany) and Fisher Scientific (Schwerte, Germany), respectively. Dichloromethane was of analytical grade (Roth, Karlsruhe, Germany). Potato dextrose agar (PDA) was purchased from Sigma-Aldrich, Steinheim, Germany. Dimethyl sulfoxide (DMSO) of bioscience grade was purchased from Roth, Karlsruhe, Germany. Dulbecco’s modified Eagle’s medium (DMEM) with 4.5 g/L glucose, 100 U/mL penicillin, 100 μg/mL streptomycin, and 10% (*v*/*v*) fetal bovine serum (FBS) was purchased from Gibco, Thermo Fisher, Darmstadt, Germany.

### Fungal material, cultivation, extraction and isolation

The fungal strain *S. chartarum* DSM 12880 (chemotype S) was purchased from Leibniz Institute DSMZ-German Collection of Microorganisms and Cell Cultures (DSMZ, Braunschweig, Germany). After reviving the freeze-dried culture according to the instruction of the DSMZ, the strain was maintained on slants of PDA medium at 4 °C. Seed cultures were prepared in Erlenmeyer flasks (500 mL) containing 200 mL of liquid PD medium on a laboratory shaker at 150 rpm at 25 °C for 3 days in darkness. The media were autoclaved at 121 °C for 15 min. Petri dishes (92 × 16 mm, Sarstedt, Nümbrecht, Germany) were prepared with PDA for main cultivation, three-point inoculated with 1 mL seed culture, and incubated for 21 days at 25 °C protected from light in an incubator.

After 21 days, the agar plates were extracted exhaustively three times using EtOAc, yielding a crude extract. Afterwards, the crude extract was filtered through Miracloth (Merck, Darmstadt, Germany) to remove spores and agar and evaporated to dryness at 40 °C using a rotary evaporator. The residue was redissolved in dichloromethane, subjected to silica gel column chromatography (CC) (Silica gel 60, 230–400 mesh, Merck, Darmstadt, Germany) and eluted applying a cyclohexane-ethyl acetate gradient of increasing polarity (from 1:0 to 0:1) with 3 mL/min. Fractions were checked by TLC, and similar fractions containing **1**–**3** were combined and further separated and purified by semi-preparative RP-HPLC eluting with 100% MeCN at 4 mL/min to yield **1** (3 mg), **2** (5 mg), and **3** (8 mg).

### Cell culture experiments

For the cytotoxicity assay, 20 mM stock solutions and 200-fold concentrations of the used concentration range (0.1–100 μM) of **1**–**3** were prepared in DMSO. These stock solutions were diluted to the respective concentrations with serum containing culture medium. Human hepatic cancer cells (HepG2, ACC 180, DSMZ, Braunschweig, Germany) were cultivated in DMEM using standardized culture conditions (37 °C, 5% CO_2_, saturated humidified atmosphere). For evaluation of cytotoxic effects, the resazurin reduction assay (Alamar Blue assay) based on O’Brien was performed in 96-well plates (O’Brien et al. 2000). Cells were treated with **1**–**3** (0.1–100 μM) and incubated for 24 h, whereas 0.01% (*w*/*v*) saponin from *Quillaja* bark (Sigma-Aldrich, Steinheim, Germany) served as positive control. Viability was calculated after blank subtraction as test over control (T/C). Six replicates of all experiments were performed for each of three independent passages (*n* = 3). The data are presented as the mean ± standard deviation (SD) in Fig. S22 (Supplementary Material). The IC_50_ and significance values were calculated with OriginPro 2016G (OriginLab Corporation, Northampton, USA): significant at *p ≤ 0.05 and highly significant at **p ≤ 0.01 and ***p ≤ 0.001.

## Results and discussion

### Isolation and structure elucidation

The filamentous fungus *S. chartarum* DSM 12880 (chemotype S) was cultured on PDA for 3 weeks and extracted with EtOAc. The crude extract was fractionated by silica gel column chromatography and semi-preparative RP-HPLC-UV to yield compounds **1**–**3**.

**Fig. 3:**
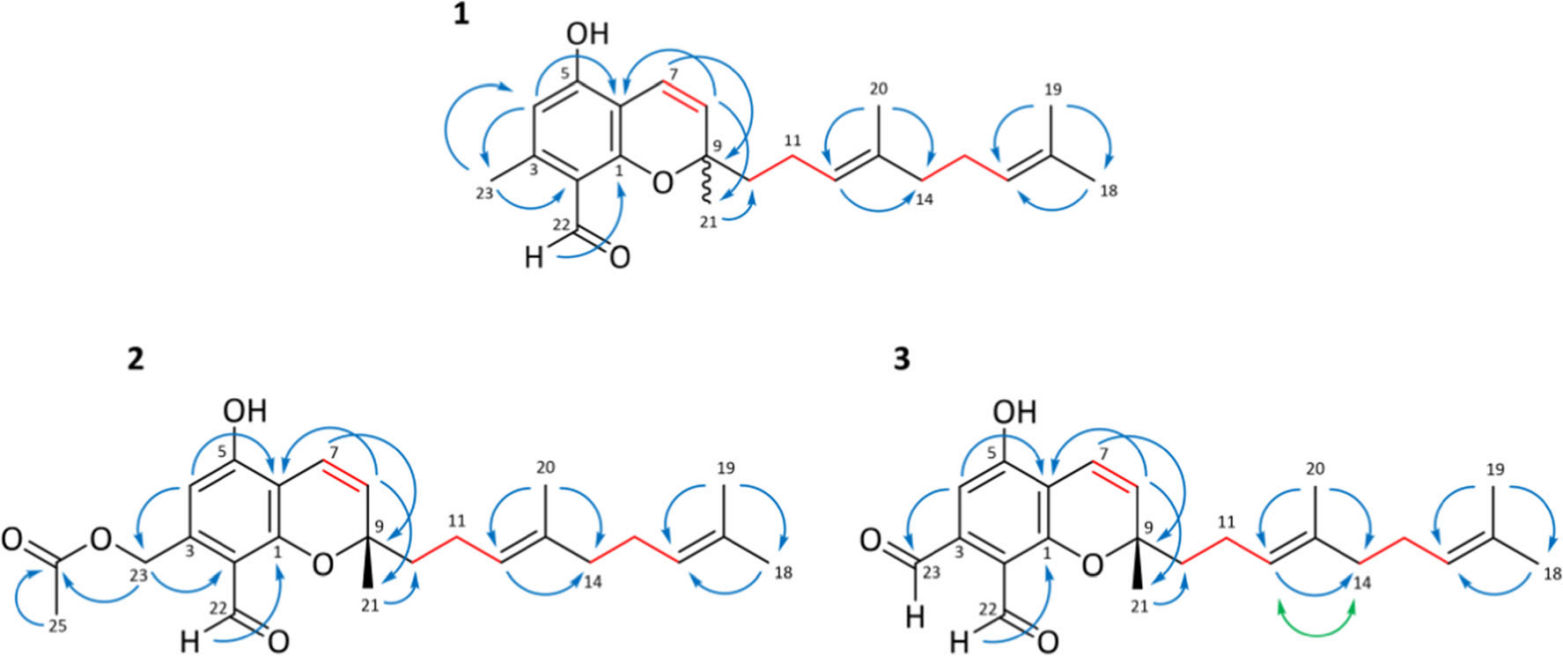
Key ^1^H-^1^H COSY (red lines), HMBC (blue →), and NOE (green ↔) correlations of **1**–**3**

Stachybotrychromene A (**1**) was isolated as a brownish oil. Its molecular formula was determined to be C_23_H_30_O_3_ by ESI-HRMS ([M-H]^−^ *m*/*z* 353.2115, calc. 353.2122), corresponding to the ^1^H and ^13^C NMR data (Table 1) and HSQC spectrum, which indicated nine degrees of unsaturation. The ^13^C and HSQC spectra revealed 23 carbon signals. Briefly, one aldehyde carbon (*δ*_C_ 195.17), six aromatic carbons (*δ*_H_ 161.6, 161.1, 145.7, 114.1, 111.6, 107.6), one oxygen-linked quaternary carbon (*δ*_C_ 81.6), four olefinic carbons (*δ*_C_ 128.0, 125.2, 124.8, 116.1), two quaternary carbons (*δ*_C_ 136.3, 132.2), four methylene groups (*δ*_C_ 42.1, 40.3, 27.3, 23.3), and five methyl groups (*δ*_C_ 27.4, 25.5, 18.4, 17.7, 16.0) (Table 1). The ^1^H NMR spectrum displayed the presence of a phenol proton at *δ*_H_ 12.76 (1H, s, OH-5), an aldehyde proton at *δ*_H_ 10.05 (1H, s, H-22), an aromatic proton at *δ*_H_ 6.20 (1H, s, H-4), and an aromatic methyl group at *δ*_H_ 2.49 (3H, s, H-23). Further examination of the spectrum disclosed a couple of *cis*-configured olefinic protons, *δ*_H_ 6.64 (1H, d, *J* = 10.2 Hz, H-7) and 5.61 (1H, d, *J* = 10.2 Hz, H-8), whereas the characteristic COSY correlation established the partial structure (Fig. 3 and Supplementary Material Fig. S3). The HMBC interactions from the olefinic proton at *δ*_H_ 5.61 (H-8) with carbons at *δ*_C_ 107.6 (C-6), 27.4 (C-21), and 42.1 (C-10) and the olefinic proton at *δ*_H_ 6.64 (H-7) with carbons at *δ*_C_ 161.1 (C-1) and 81.6 (C-9) confirmed a chromene moiety attached to a side chain. According to the ^1^H NMR data, this side chain contains four methyl groups; one of them directly linked between the chromene ring and the side chain (*δ*_H_ 1.39 (3H, s, H-21)) as confirmed by HMBC experiments as well. The three remaining methyl groups at *δ*_H_ 1.54 (3H, s, H-20), *δ*_H_ 1.57 (3H, s, H-18), and *δ*_H_ 1.64 (3H, s, H-19), in addition to the two deshielded CH triplets at *δ*_H_ 5.11 (1H, t, *J* = 7.2 Hz, H-12) and *δ*_H_ 5.07 (1H, t, *J* = 7.0 Hz, H-16), imply the presence of an isoprene unit. As assigned from COSY and HMBC resonances, the remaining four methylene signals at *δ*_H_ 1.69 (2H, m, H-10), *δ*_H_ 2.07 (2H, m, H-11), *δ*_H_ 1.94 (2H, m, H-14, overlapped by solvent), and *δ*_H_ 2.03 (2H, m, H-15) completed the structural features of the two prenyl groups derived from the isoprene side chain and concluded the presence of 32 protons in total. The determination of the absolute configuration was not possible, most likely due to tiny impurities adversely affecting the CD properties of the molecule. The compound was designated to be (*E*)-2-(4,8-dimethylnona-3,7-dien-1-yl)-5-hydroxy-2,7-dimethyl-2H-chromene-8-carbaldehyde.

**Table 1:** ^1^H and ^13^C NMR data of stachybotrychromenes A–C (**1**–**3**) in CD_3_CN (*δ* in ppm, *J* in Hz)

	**1**		**2**		**3**	
No.	*δ*_C_, type	*δ*_H_ (*J* in Hz)	*δ*_C_, type	*δ*_H_ (*J* in Hz)	*δ*_C_, type	*δ*_H_ (*J* in Hz)
1	161.1, C		161.2, C		161.5, C	
2	114.1, C		115.9, C		113.1, C	
3	145.7, C		142.0, C		138.8, C	
4	111.6, CH	6.20, s	111.3, CH	6.40, s	117.5, CH	6.90, s
5	161.6, C		161.2, C		160.4, C	
6	107.6, C		109.6, C		114.0, C	
7	116.1, CH	6.64, d (10.2)	113.1, CH	6.66, d (10.2)	115.8, CH	6.71, d (10.2)
8	128.0, CH	5.61, d (10.2)	129.1, CH	5.67, d (10.2)	131.8, CH	5.82, d (10.2)
9	81.6, C		82.0, C		82.4, C	
10	42.1, CH_2_	1.69, m	42.1, CH_2_	1.72, m	42.1, CH_2_	1.77, m
11	23.3, CH_2_	2.07, m	23.3, CH_2_	2.09, m	23.2, CH_2_	2.12, m
12	124.8, CH	5.11, t (7.2)	124.7, CH	5.12, t (7.2)	124.6, CH	5.12, t (7.2)
13	136.3, C		136.3, C		136.5, C	
14	40.3, CH_2_	1.94, m	40.3, CH_2_	1.93, m	40.3, CH_2_	1.94, m
15	27.3, CH_2_	2.03, m	27.3, CH_2_	2.02, m	27.3, CH_2_	2.03, m
16	125.2, CH	5.07, t (7.0)	125.2, CH	5.07, t (7.0)	125.2, CH	5.07, t (7.0)
17	132.2, C		132.2, C		132.2, C	
18	17.7, CH_3_	1.57, s	17.8, CH_3_	1.57, s	17.7, CH_3_	1.57, s
19	25.8, CH_3_	1.64, s	25.8, CH_3_	1.64, s	25.8, CH_3_	1.64, s
20	16.0, CH_3_	1.54, s	16.0, CH_3_	1.54, s	16.0, CH_3_	1.54, s
21	27.4, CH_3_	1.39, s	27.5, CH_3_	1.41, s	27.5, CH_3_	1.45, s
22	195.2, CHO	10.05, s	194.9, CHO	10.01, s	196.6, CHO	10.63, s
23	18.4, CH_3_	2.49, s	63.3, CH_2_	5.26, s	193.5, CHO	10.02, s
24			171.2, C			
25			21.1, CH_3_	2.04, s		
OH-5		12.76, s		12.72, s		12.95, s

The molecular formula of stachybotrychromene B (**2**) was in accordance with C_25_H_32_O_5_ (ESI-HRMS, [M-H]^−^ *m*/*z* 411.2177 (calc. *m*/*z* 411.2175)) and the compound was obtained as brownish oil. The degree of unsaturation was designated to be 10. The analysis of 1D and 2D NMR (HSQC, COSY, and HMBC) data proved **2** to have a similar core structure as described for **1**, with the exception of an acetyl group (*δ*_C_ 171.2 (C-24), *δ*_C_ 21.1 (C-25)/*δ*_H_ 2.04, 3H, s, H-25) replacing the methyl group at C-23 of **1**. The downfield shift of the methylene protons (*δ*_H_ 5.26, 2H, s, H-23) and the respective carbon shift (*δ*_C_ 63.3, C-23) suggest the attachment of the acetyl group to C-23 via an ester bond (Table 1). HMBC resonances between H-25 and C-24, H-23 and C-24, H-4 and C-23, and H-23 and C-2 confirmed the assignment (Fig. 3). Lastly, an in-source acetate loss (*m*/*z* 351.1960 [M-CH_3_COOH]^−^) observed in the ESI-HRMS spectrum established the structure unequivocally (Supplementary Material Fig. S12).

**Fig. 4:**
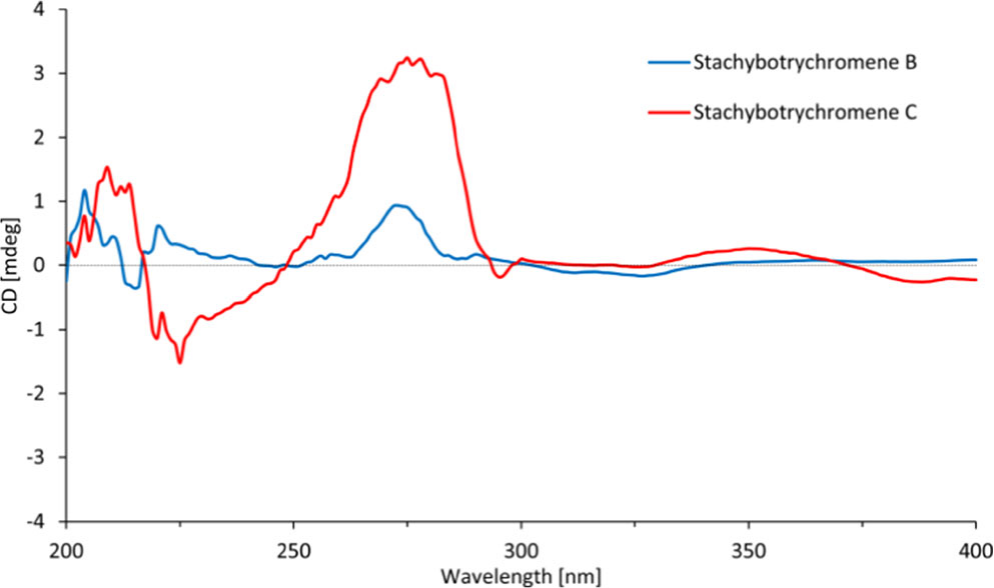
CD spectra of **2** and **3**

The absolute configuration of the stereocenter at C-9 was determined as (*S*) based on the weak but characteristic positive Cotton effect at 260–290 nm observed in the CD spectrum (Fig. 4), which is in agreement with reported analogues (Li et al. 2014b; Bao et al. 2015; Zhang et al. 2017). Therefore, **2** was identified to be (*S*, *E*)-(2-(4,8-dimethylnona-3,7-dien-1-yl)-8-formyl-5-hydroxy-2-methyl-2*H*-chromen-7-yl)methyl acetate.

Stachybotrychromene C (**3**) was obtained as a yellow oil, and its molecular formula was determined to be C_23_H_28_O_4_ based on ESI-HRMS ([M-H]^−^ *m*/*z* 367.1915, calc. 367.1907) and NMR data (Table 1), corresponding to 10 degrees of unsaturation. The ^1^H, ^13^C, HSQC, COSY, and HMBC spectroscopic data are highly comparable to those of **1** and **2**, except for the additional aldehyde group with corresponding signals at *δ*_H_ 10.02 (1-H, s, H-23) and *δ*_C_ 193.5 (C-23) instead of a methyl or acetoxymethylene group, respectively.

Although the chemical shifts of the increment system of the prenyl scaffold of the H-12 proton at *δ*_H_ 5.11 and the C-20 signal *δ*_C_ 16.00 (analogously to **1** and **2**, Table 1) already indicate *E* configuration (Nishino and Bowers 1976; Ma et al. 2015), the configuration of the isoprene unit of **3**, as representative example, was in addition investigated by extensive and selective 1D and 2D NOE experiments. Moreover, the herein presented compounds **1**–**3** are closely related to the recently published stachybonoids A–C (Zhang et al. 2017), which were interestingly described to possess a *cis*-configured isoprene unit. However, selective NOE experiments with saturation on the proton of the C-12 (*δ*_H_ 5.12) enhanced the methylene unit at *δ*_H_ 1.94 (H-14) (Fig. 3 and Supplementary Material Fig. S18 and Fig. S19), thus confirming the *E* configuration of the double bond between C-12 and C-13.

The absolute configuration of the C-9 was also assigned as (*S*) based on CD measurement as described above (Fig. 4). Hence, the structure was determined to be (*S*, *E*)-2-(4,8-dimethylnona-3,7-dien-1-yl)-5-hydroxy-2-methyl-2*H*-chromene-7,8-dicarbaldehyde.

### Proposed biosynthesis of **1**–**3** and occurrence in other *Stachybotrys* strains

Meroterpenoids are hybrid biosynthesized natural products, partially derived from terpenoids (Geris and Simpson 2009). Regarding compounds **1**–**3**, this hybrid structure is comprised of an additional tetraketide part. Key intermediate is *o*-orsellinic acid (OA), which is formed from acetyl-CoA and 3 units of malonyl-CoA after cyclization. Afterwards, a prenylation step leads to the attachment of the prenyl side chain and the carboxy function of OA undergoes reduction to an aldehyde group to form LL-Z1272β, also known as ilicicolin B (Li et al. 2016). This compound is well known as a key intermediate within the biosynthetic pathway of many meroterpenoids and is therefore assumed to be the precursor for compounds **1**–**3**.

Ultimately, **1** bears the aromatic methyl group at C-23, derived from *o*-orsellinaldehyde, which is further transformed into an oxidized acetoxymethylene group yielding **2**. Subsequently, the final oxidation at C-23 leads to the formation of **3**, possessing the respective aldehyde group. HPLC-MS/MS analysis of the metabolic profile of *S. chartarum* (DSM 12880) showed that **1** and **2** are the main compounds formed during the first days of incubation. The signals of **1** and **2** decrease over time and **3** occurs as the major compound at later time points (data not shown). Thus, **1** and **2** are most likely intermediates, whereas **3** is the final product within this biosynthetic pathway. These observations are in agreement with reported (proposed) biosynthetic pathways of other meroterpenoids among the genus *Stachybotrys* (Zhang et al. 2017; Zhao et al. 2017c).

**1**–**3** are produced by a diversity of *Stachybotrys* strains. Within metabolite profiling, it was observed that also *S. chartarum* chemotype A (IBT 40288, CBS 109291), S (IBT 40293, CBS 109290), and *Stachybotrys chlorohalonata* (IBT 40285, CBS 109283) are capable producers, as well on different media, such as malt extract agar. Only *S. chartarum* DSMZ 63425 appeared with a different profile, and no stachybotrychromenes were detected (data not shown).

### Cytotoxicity

For assessment of cytotoxic effects, compounds **1**–**3** were tested on HepG2 cells, using the resazurin reduction assay. For compound **1**, the results indicated that cell viability is significantly reduced with treatment of 50 μM (*p* < 0.05) after 24 h of incubation. Compound **2** was more potent and showed significantly reduced cell survival rates at 25 μM (p < 0.05), whereas for compound **3**, no significantly reduced cell viability up to 100 μM was observed (Supplementary Material, Fig. S22). For **1** and **2**, IC_50_ values were determined to be 73.7 and 28.2 μM, respectively.

Due to the fact that chromenes and chromanes have been reported to possess antiprotozoal and antiviral properties (Harel et al. 2013; Li et al. 2014b; Presley et al. 2017), further investigations regarding those biological activities of the newly isolated compounds might be of interest.

The present work discovered three novel meroterpenoid derivatives (**1**–**3**), named stachybotrychromenes A–C, isolated from *S. chartarum* DSM 12880 (chemotype S) containing a chromene ring backbone structure with an attached isoprenoid side chain. In view of the biosynthetic pathway, it can be assumed that **3** is the final product and is biosynthesized via the intermediates **1** and **2**. In terms of cytotoxicity, **1** and **2** showed moderate cytotoxic effects on HepG2 cells after 24 h of exposure, whereas **3** exhibited no cytotoxicity within the investigated concentration range (0.1–100 μM). It should be noticed that these compounds are produced by both chemotypes of *S. chartarum* as well as *S. chlorohalonata* and contribute to the toxic profile of this genus.

## Electronic supplementary material

ESM 1 (PDF 2.80 MB)

## References

[CR1] Andersen B, Nielsen KF, Thrane U, Szaro T, Taylor JW, Jarvis BB (2003). Molecular and phenotypic descriptions of *Stachybotrys chlorohalonata* sp. nov. and two chemotypes of *Stachybotrys chartarum* found in water-damaged buildings. Mycologia.

[CR2] Bao Y-R, Chen G-D, Wu Y-H, Li X-X, Hu D, Liu X-Z, Li Y, Yao X-S, Gao H (2015). Stachybisbins A and B, the first cases of seco-bisabosquals from *Stachybotrys bisbyi*. Fitoterapia.

[CR3] Bisby GR (1943). Stachybotrys. Trans Br Mycol Soc.

[CR4] Bloom E, Grimsley LF, Pehrson C, Lewis J, Larsson L (2009). Molds and mycotoxins in dust from water-damaged homes in New Orleans after hurricane Katrina. Indoor Air.

[CR5] Chunyu W-X, Ding Z-G, Li M-G, Zhao J-Y, Gu S-J, Gao Y, Wang F, Ding J-H, Wen M-L (2016). Stachartins A–E, phenylspirodrimanes from the tin mine tailings-associated fungus *Stachybotrys chartarum*. Helv Chim Acta.

[CR6] Dearborn DG, Yike I, Sorenson WG, Miller MJ, Etzel RA (1999). Overview of investigations into pulmonary hemorrhage among infants in Cleveland. Ohio Environ Health Perspect.

[CR7] Ding Z-G, Ding J-H, Zhao J-Y, Chunyu W-X, Li M-G, Gu S-J, Wang F, Wen M-L (2017). A new phenylspirodrimane dimer from the fungus *Stachybotrys chartarum*. Fitoterapia.

[CR8] Ding Z-G, Zhao J-Y, Ding J-H, Chunyu W-X, Li M-G, Gu S-J, Wang F, Wen M-L (2017b) A novel phenylspirodrimane dimer from cultures of the fungus *Stachybotrys chartarum*. Nat Prod Res:1–5. 10.1080/14786419.2017.1413565 10.1080/14786419.2017.1413565 29252003

[CR9] Etzel RA, Montaña E, Sorenson WG, Kullman GJ, Allan TM, Dearborn DG (1998). Acute pulmonary hemorrhage in infants associated with exposure to Stachybotrys atra and other fungi. Arch Pediatr Adolesc Med.

[CR10] Geris R, Simpson TJ (2009). Meroterpenoids produced by fungi. Nat Prod Rep.

[CR11] Harel D, Schepmann D, Prinz H, Brun R, Schmidt TJ, Wünsch B (2013). Natural product derived antiprotozoal agents: synthesis, biological evaluation, and structure-activity relationships of novel chromene and chromane derivatives. J Med Chem.

[CR12] Hasumi K, Hasegawa K, Kitano Y (2007). Isolation and absolute configuration of SMTP-0, a simplest congener of the SMTP family nonlysine-analog plasminogen modulators. J Antibiot.

[CR13] Hossain MA, Ahmed MS, Ghannoum MA (2004). Attributes of *Stachybotrys chartarum* and its association with human disease. J Allergy Clin Immunol.

[CR14] Jarvis BB (2003). *Stachybotrys chartarum*: a fungus for our time. Phytochemistry.

[CR15] Jarvis BB, Salemme J, Morals A (1995). *Stachybotrys* toxins. 1. Nat Toxins.

[CR16] Jarvis BB, Sorenson WG, Hintikka EL, Nikulin M, Zhou Y, Jiang J, Wang S, Hinkley S, Etzel RA, Dearborn D (1998). Study of toxin production by isolates of *Stachybotrys chartarum* and *Memnoniella echinata* isolated during a study of pulmonary hemosiderosis in infants. Appl Environ Microbiol.

[CR17] Jarvis B, Hinkley S, Nielsen K (2000). Stachybotrys: an unusual mold associated with water-damaged buildings. Mycotoxin Res.

[CR18] Johanning E, Biagini R, Hull D, Morey P, Jarvis B, Landsbergis P (1996). Health and immunology study following exposure to toxigenic fungi (*Stachybotrys chartarum*) in a water-damaged office environment. Int Arch Occup Environ Health.

[CR19] Li Y, Wu C, Liu D, Proksch P, Guo P, Lin W (2014). Chartarlactams A-P, phenylspirodrimanes from the sponge-associated fungus *Stachybotrys chartarum* with antihyperlipidemic activities. J Nat Prod.

[CR20] Li Y, Liu D, Cen S, Proksch P, Lin W (2014). Isoindolinone-type alkaloids from the sponge-derived fungus *Stachybotrys chartarum*. Tetrahedron.

[CR21] Li C, Matsuda Y, Gao H, Hu D, Yao XS, Abe I (2016). Biosynthesis of LL-Z1272β: discovery of a new member of NRPS-like enzymes for aryl-aldehyde formation. Chembiochem.

[CR22] Li W, Yang Y-B, Yang X-Q, Xie H-D, Shao Z-H, Zhou H, Miao C-P, Zhao L-X, Ding Z-T (2017). Novel isochroman dimers from Stachybotrys sp. PH30583: fermentation, isolation, structural elucidation and biological activities. Planta Med.

[CR23] Liu D, Li Y, Li X, Cheng Z, Huang J, Proksch P, Lin W (2017). Chartarolides A-C, novel meroterpenoids with antitumor activities. Tetrahedron Lett.

[CR24] Ma X, Wang H, Li F, Zhu T, Gu Q, Li D (2015). Stachybotrin G, a sulfate meroterpenoid from a sponge derived fungus *Stachybotrys chartarum* MXH-X73. Tetrahedron Lett.

[CR25] Matsuda Y, Abe I (2016). Biosynthesis of fungal meroterpenoids. Nat Prod Rep.

[CR26] Nishino C, Bowers WS (1976). The stereoisomers of 3,7,11-trimethyldodeca-2,6,10-triene. Tetrahedron.

[CR27] O'Brien J, Wilson I, Orton T, Pognan F (2000). Investigation of the Alamar Blue (resazurin) fluorescent dye for the assessment of mammalian cell cytotoxicity. Eur J Biochem.

[CR28] Page EH, Trout DB (2001). The role of Stachybotrys mycotoxins in building-related illness. AIHAJ - American Industrial Hygiene Association.

[CR29] Pestka JJ, Yike I, Dearborn DG, Ward MDW, Harkema JR (2008). *Stachybotrys chartarum*, trichothecene mycotoxins, and damp building-related illness: new insights into a public health enigma. Toxicol Sci.

[CR30] Presley CC, Valenciano AL, Fernández-Murga ML, Du Y, Shanaiah N, Cassera MB, Goetz M, Clement JA, Kingston DGI (2017) Antiplasmodial Chromanes and Chromenes from the monotypic plant species *Koeberlinia spinosa*. J Nat Prod. 10.1021/acs.jnatprod.7b00579 10.1021/acs.jnatprod.7b00579 PMC5866173 29048892

[CR31] Sasaoka M, Wada Y, Hasumi K (2007). Stachybotrydial selectively enhances fibrin binding and activation of Glu-plasminogen. J Antibiot.

[CR32] Sawadjoon S, Kittakoop P, Isaka M, Kirtikara K, Madla S, Thebtaranonth Y (2004). Antiviral and antiplasmodial spirodihydrobenzofuran terpenes from the fungus *Stachybotrys nephrospora*. Planta Med.

[CR33] Wang A, Xu Y, Gao Y, Huang Q, Luo X, An H, Dong J (2015). Chemical and bioactive diversities of the genera *Stachybotrys* and *Memnoniella* secondary metabolites. Phytochem Rev.

[CR34] Yin Y, Fu Q, Wu W, Cai M, Zhou X, Zhang Y (2017) Producing novel fibrinolytic isoindolinone derivatives in marine fungus *Stachybotrys longispora* FG216 by the rational supply of amino compounds according to its biosynthesis pathway. Mar Drugs 15. 10.3390/md15070214 10.3390/md15070214 PMC5532656 28678182

[CR35] Zhang P, Li Y, Jia C, Lang J, Niaz S-I, Li J, Yuan J, Yu J, Chen S, Liu L (2017). Antiviral and anti-inflammatory meroterpenoids: Stachybonoids A–F from the crinoid-derived fungus *Stachybotrys chartarum* 952. RSC Adv.

[CR36] Zhao J, Feng J, Tan Z, Liu J, Zhang M, Chen R, Xie K, Chen D, Li Y, Chen X, Dai J (2017). Bistachybotrysins A-C, three phenylspirodrimane dimers with cytotoxicity from *Stachybotrys chartarum*. Bioorg Med Chem Lett.

[CR37] Zhao J, Liu J, Shen Y, Tan Z, Zhang M, Chen R, Zhao J, Zhang D, Yu L, Dai J (2017). Stachybotrysams A–E, prenylated isoindolinone derivatives with anti-HIV activity from the fungus *Stachybotrys chartarum*. Phytochem Lett.

[CR38] Zhao J, Feng J, Tan Z, Liu J, Zhao J, Chen R, Xie K, Zhang D, Li Y, Yu L, Chen X, Dai J (2017). Stachybotrysins A-G, phenylspirodrimane derivatives from the fungus *Stachybotrys chartarum*. J Nat Prod.

