# Supplementary material for: Stachybotrychromenes A–C: novel cytotoxic meroterpenoids from Stachybotrys sp
Source: Mycotoxin Res. 2018 Mar 16;34(3):179–85. doi: 10.1007/s12550-018-0312-7 (PMC6061235; doi:10.1007/s12550-018-0312-7)
Supplement: Supplementary file 1 — (PDF 2.80 MB) [file 12550_2018_312_MOESM1_ESM.pdf]

SUPPLEMENTARY MATERIAL

Stachybotrychromenes A-C: novel cytotoxic meroterpenoids from *Stachybotrys* sp.

Annika Jagels[1], Yannick Hövelmann[1], Alexa Zielinski[1], Melanie Esselen[1], Jens Köhler[2], Florian Hübner[1], and Hans-Ulrich Humpf[1]*

[1]Institute of Food Chemistry, Westfälische Wilhelms-Universität Münster, Corrensstraße 45, 48149 Münster, Germany; [2]Institute of Pharmaceutical and Medicinal Chemistry, Corrensstraße 48, 48149 Münster, Germany.

* **Corresponding author**: Prof. Dr. Hans-Ulrich Humpf; humpf@wwu.de; Fax.: +49 251 83-33396; Telephone: +49 251 83-33391; ORCID-ID: 0000-0003-3296-3058

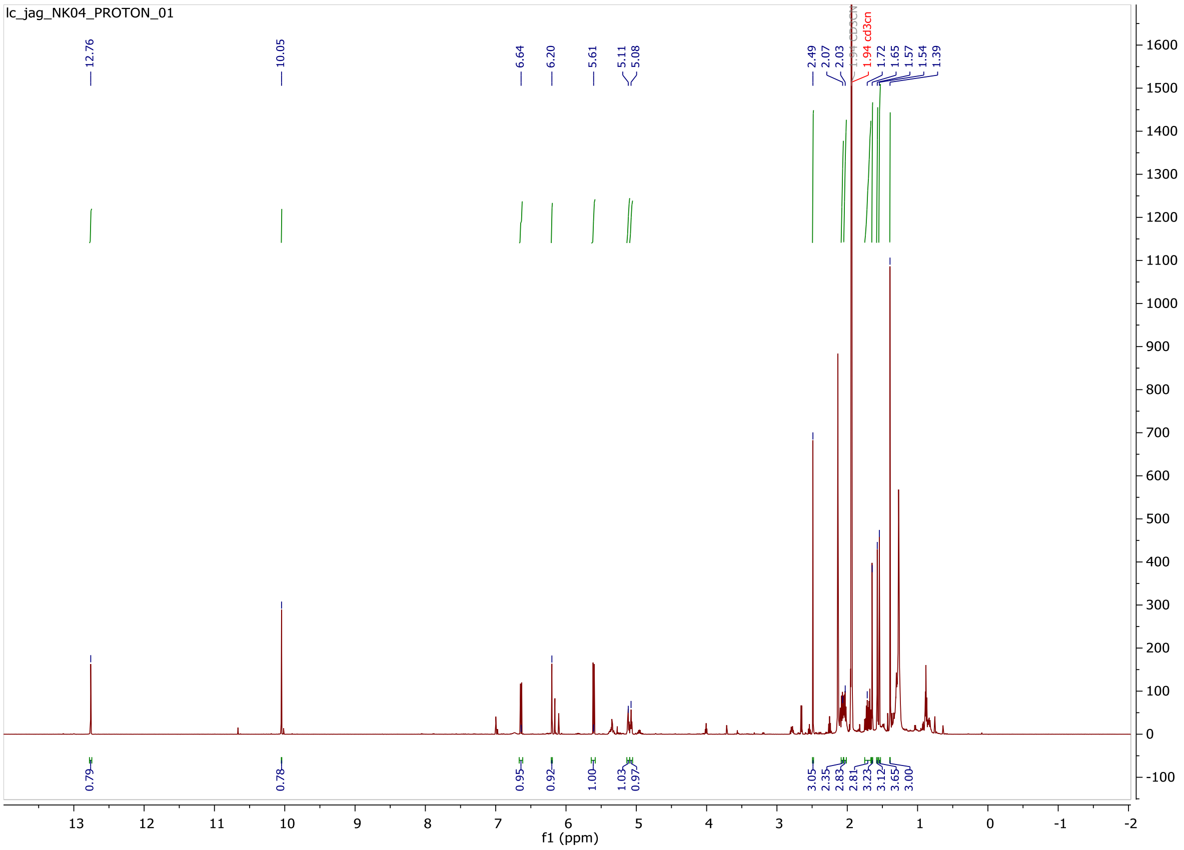

Fig. S1 ^{1}H (600 MHz) spectrum of **1** in CD_3CN.

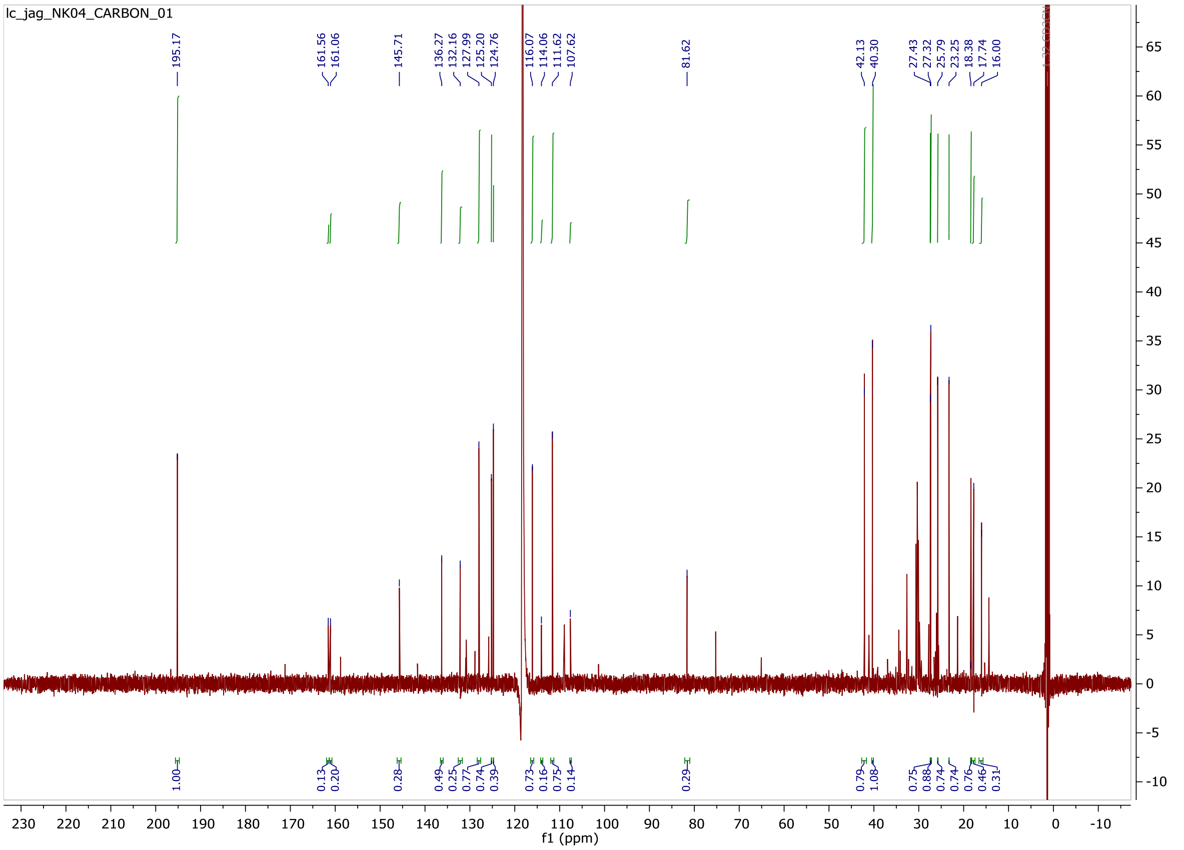

Fig. S2 ^{13}C (150 MHz) spectrum of **1** in CD_3CN.

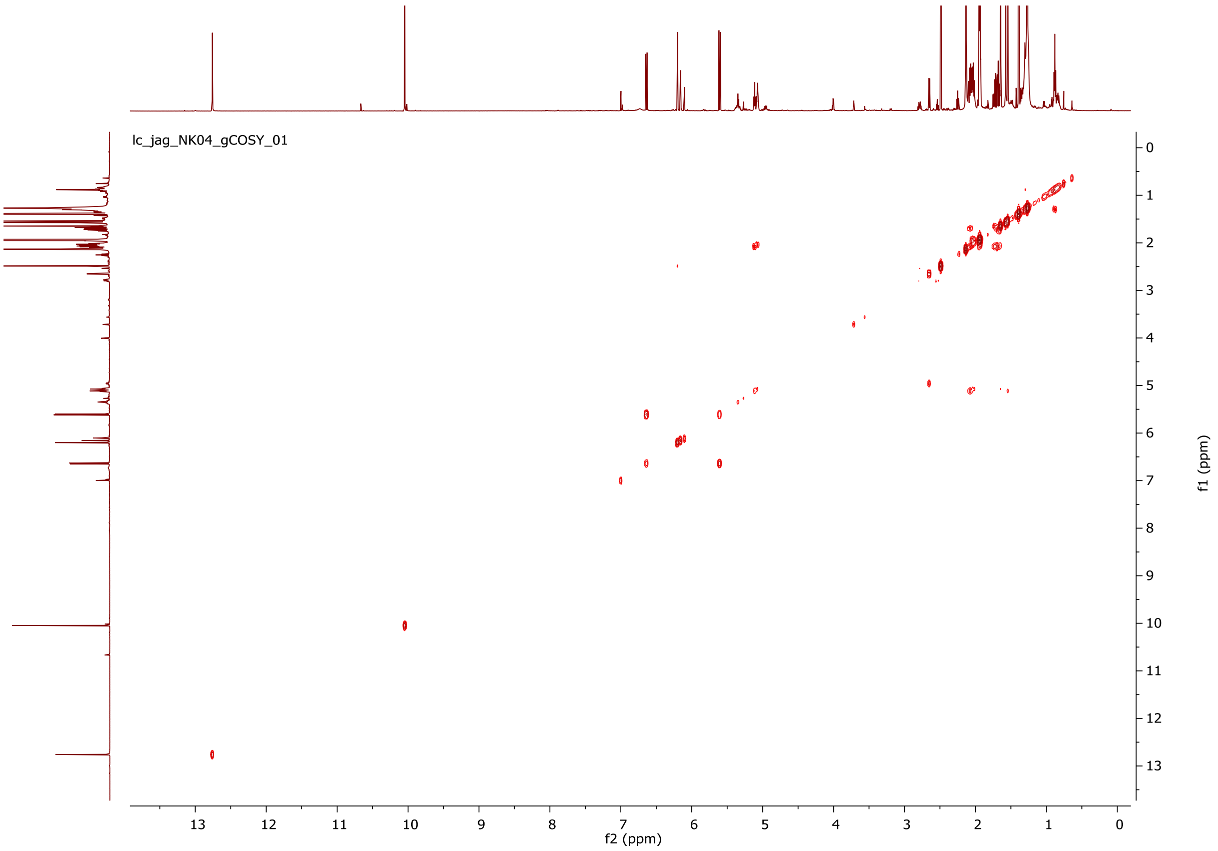

Fig. S3 ^{1}H-^{1}H COSY spectrum of **1** in CD_3CN.

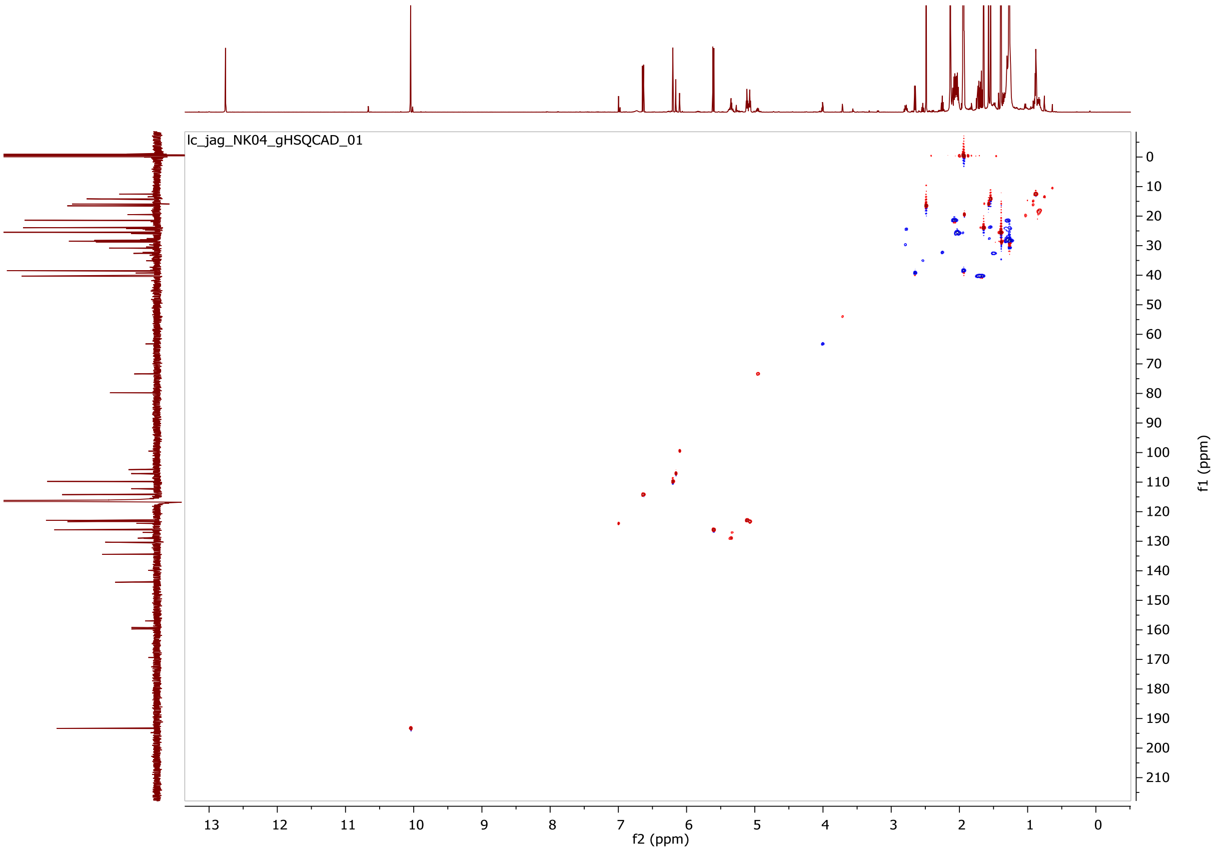

Fig. S4 HSQC spectrum of **1** in CD_3CN.

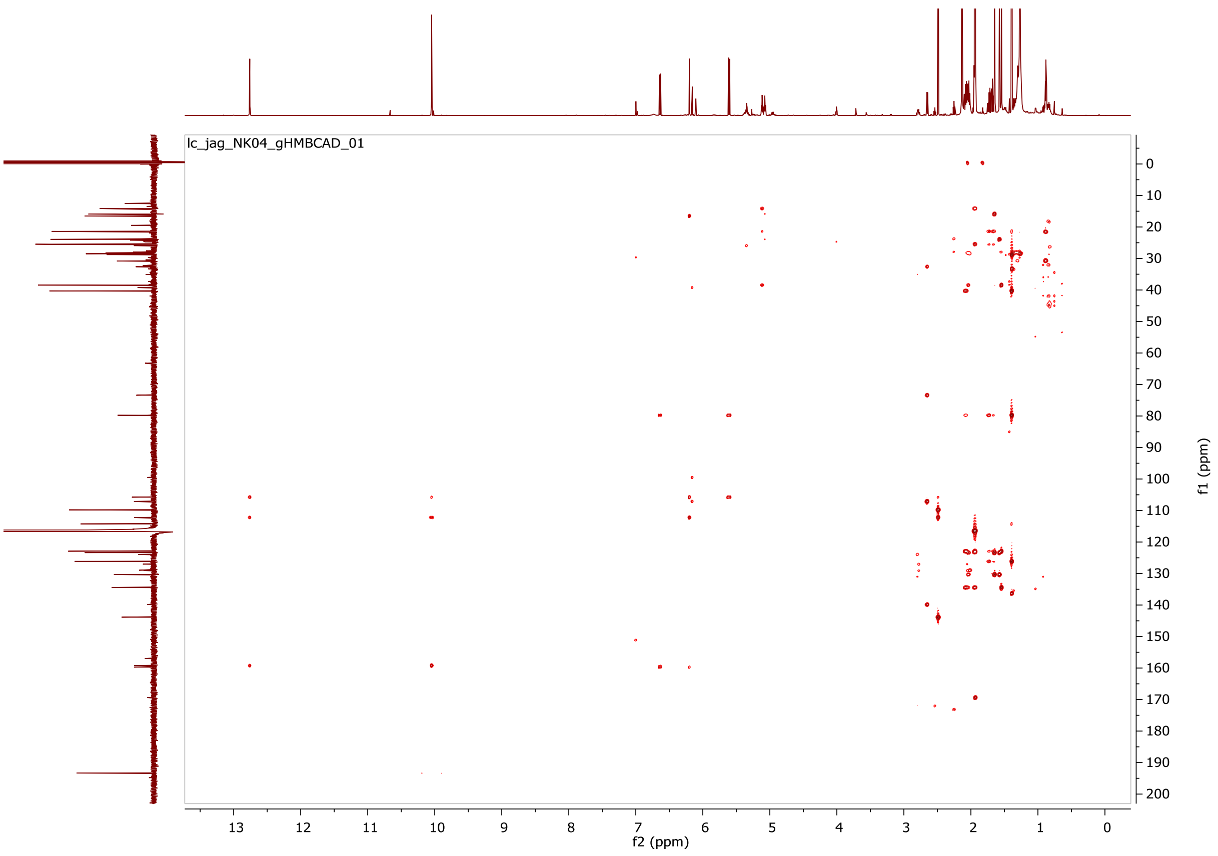

Fig. S5 HMBC spectrum of **1** in CD_3CN.

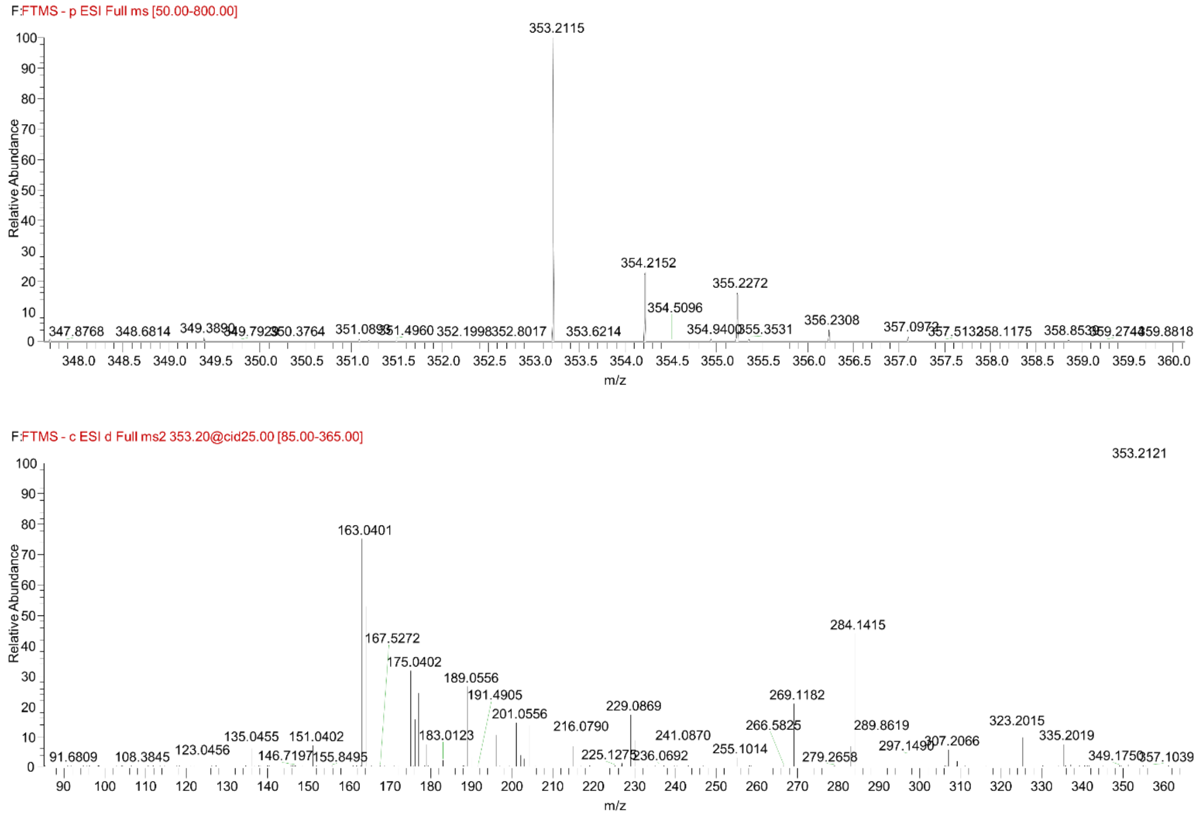

Fig. S6 HR-ESI-MS and fragmentation spectra of **1**.

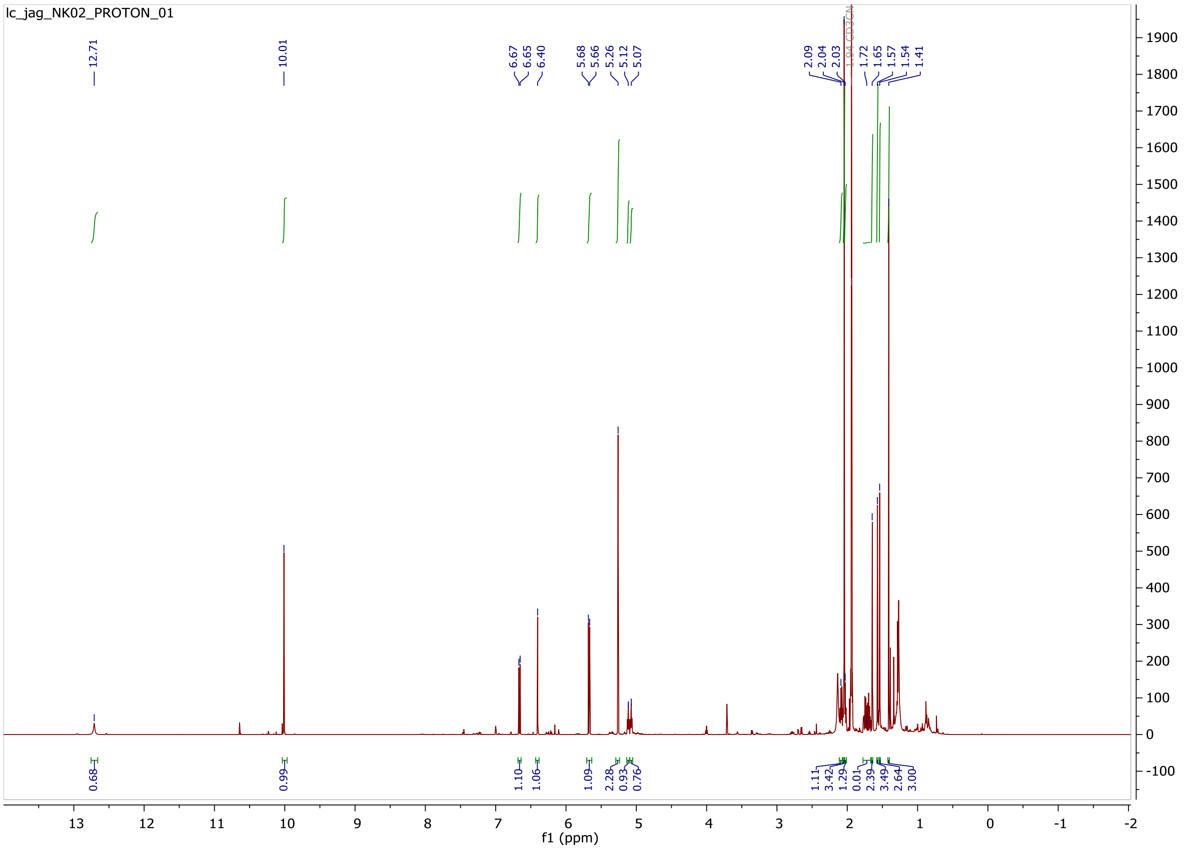

Fig. S7 ^{1}H (600 MHz) spectrum of **2** in CD_3CN.

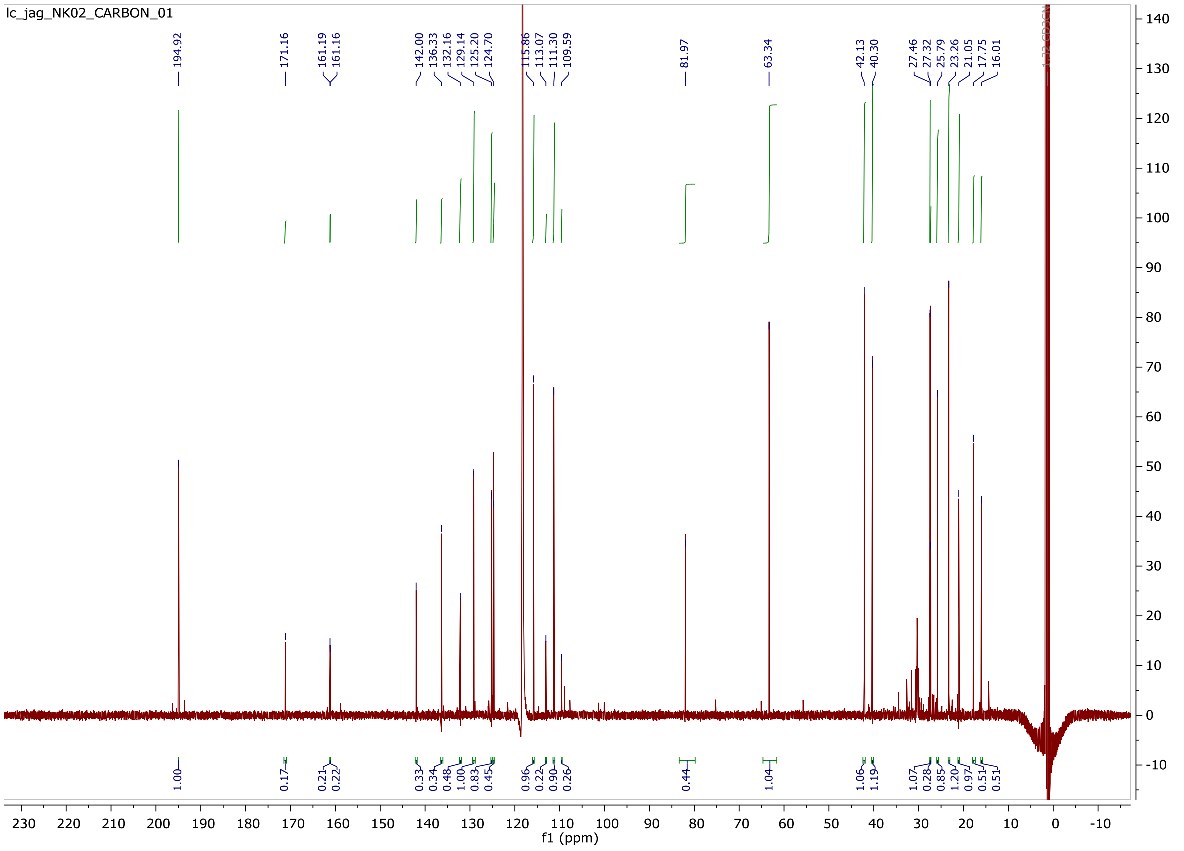

Fig. S8 ^{13}C (150 MHz) spectrum of **2** in CD_3CN.

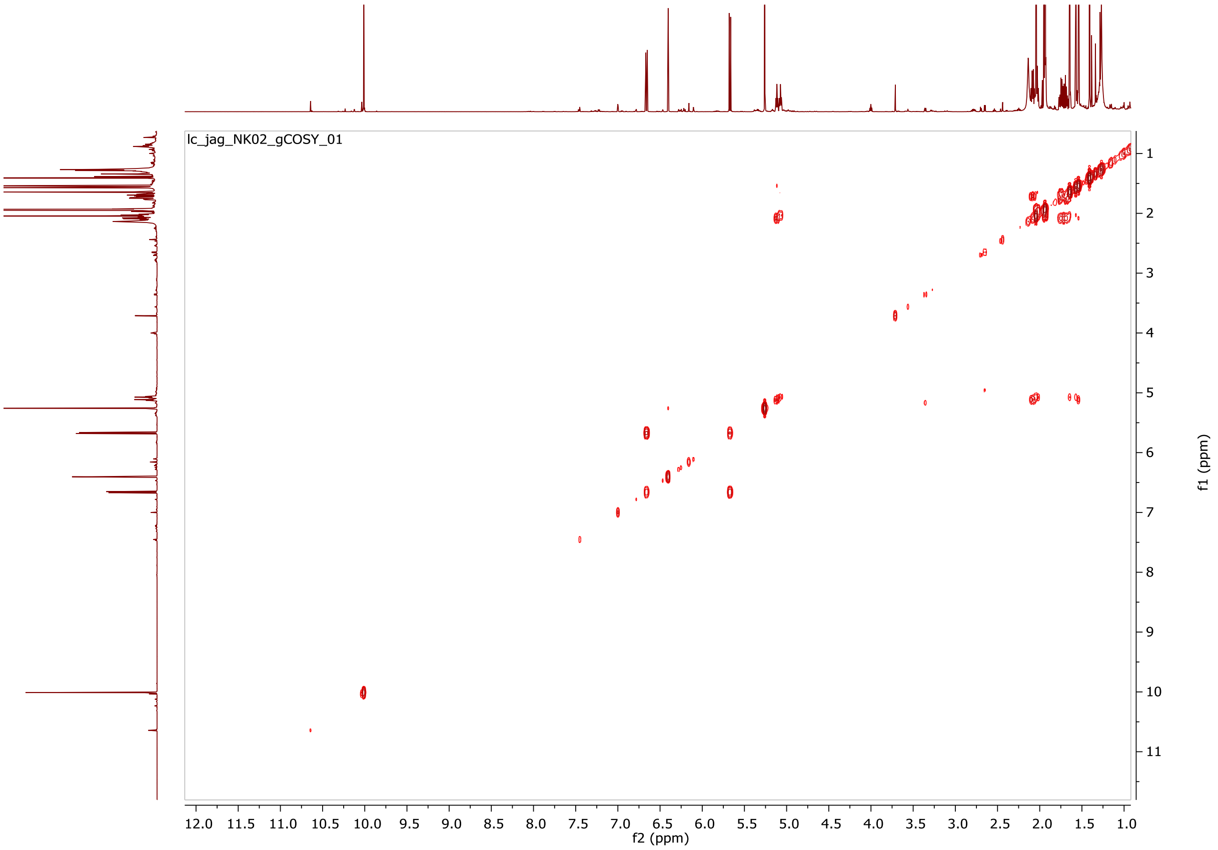

Fig. S9 ^{1}H-^{1}H COSY spectrum of **2** in CD_3CN.

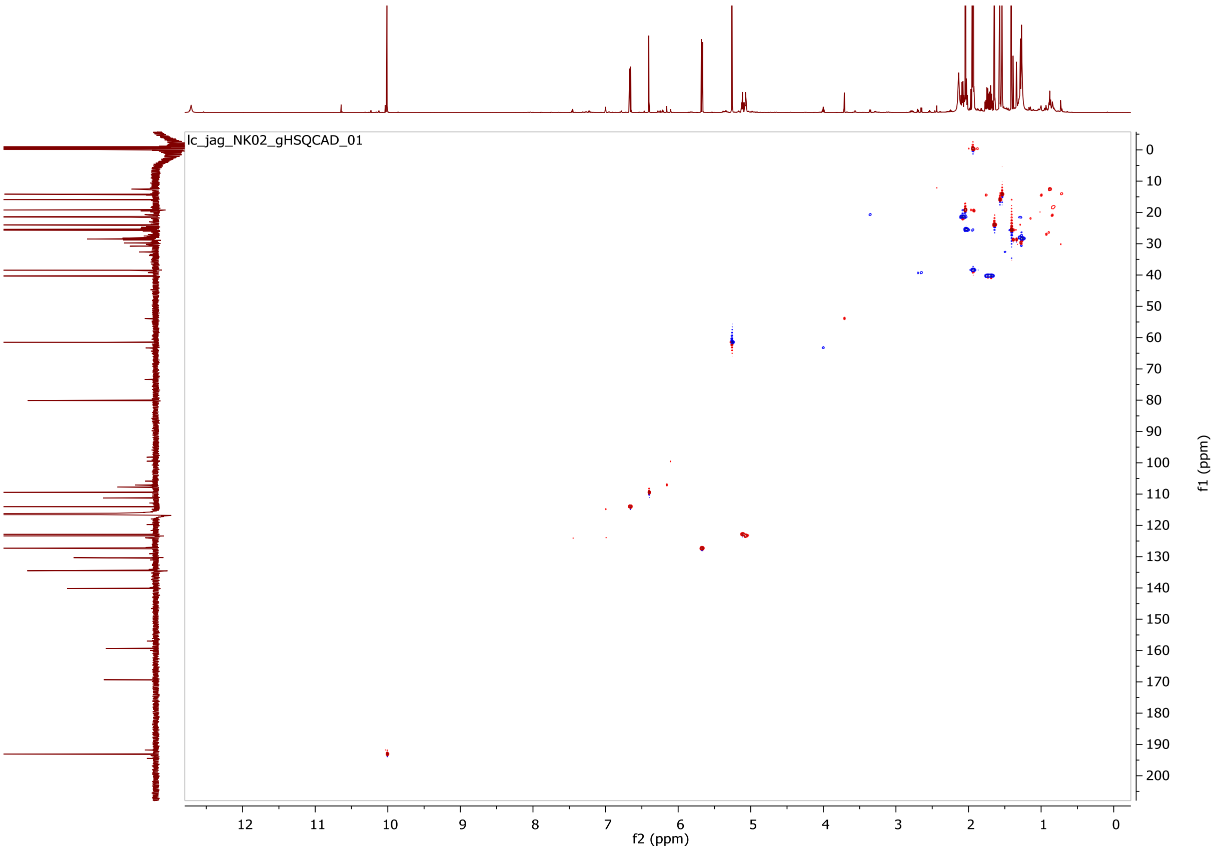

Fig. S10 HSQC spectrum of **2** in CD_3CN.

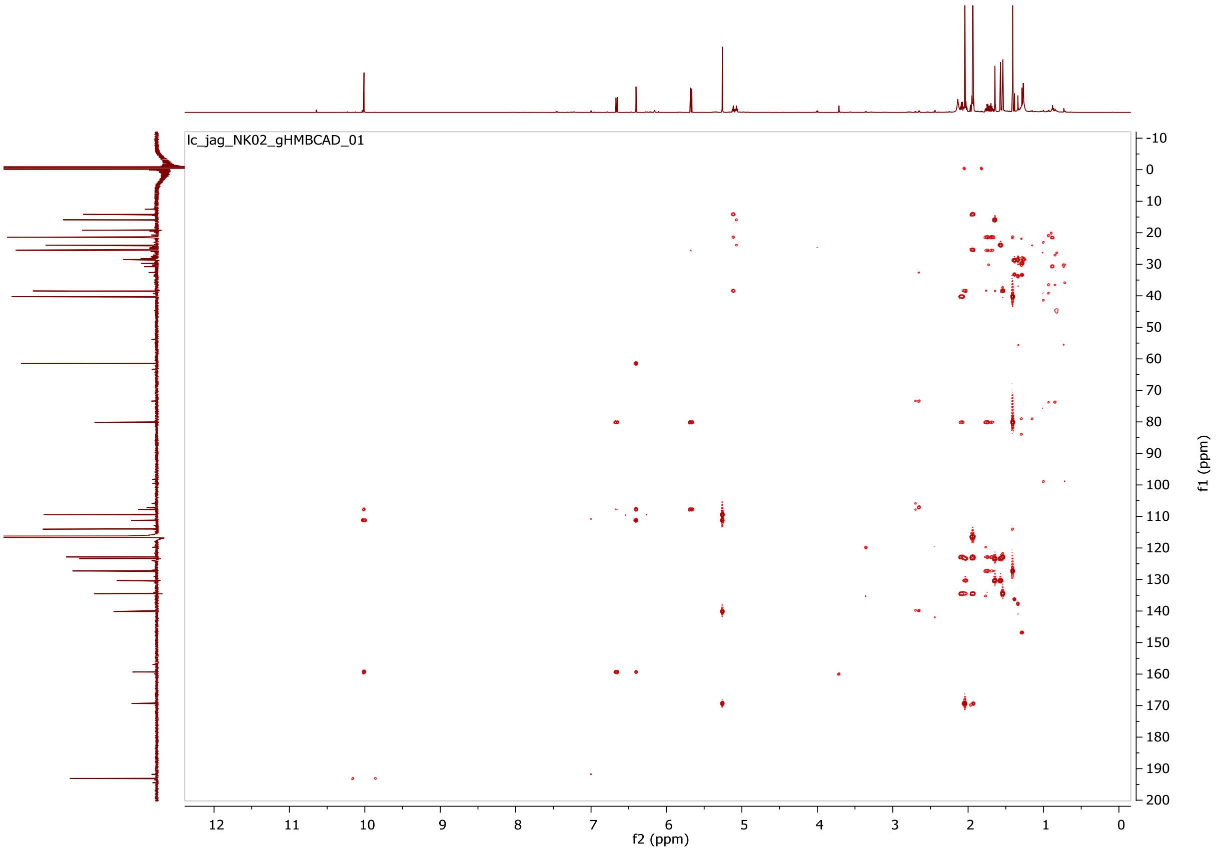

Fig. S11 HMBC spectrum of **2** in CD_3CN.

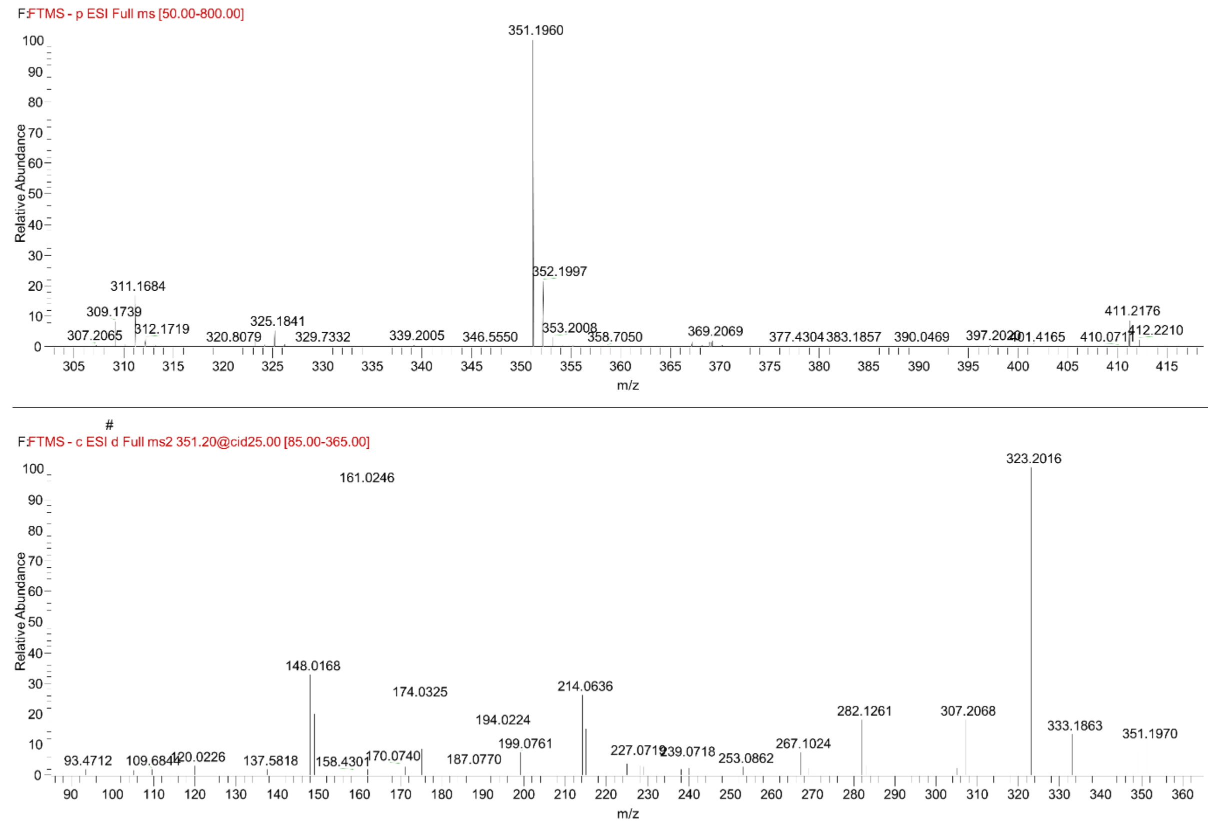

Fig. S12 HR-ESI-MS and fragmentation spectra of **2**.

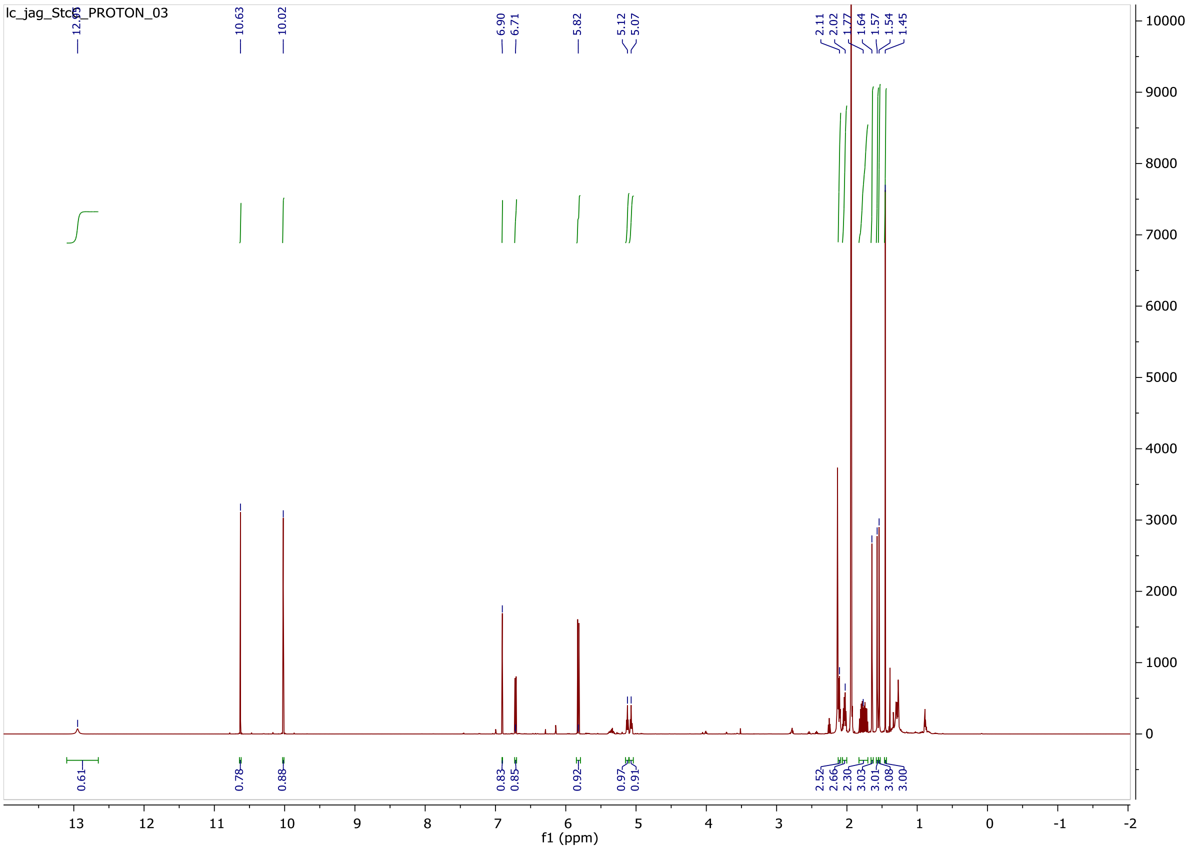

Fig. S13 ^{1}H (600 MHz) spectrum of **3** in CD_3CN.

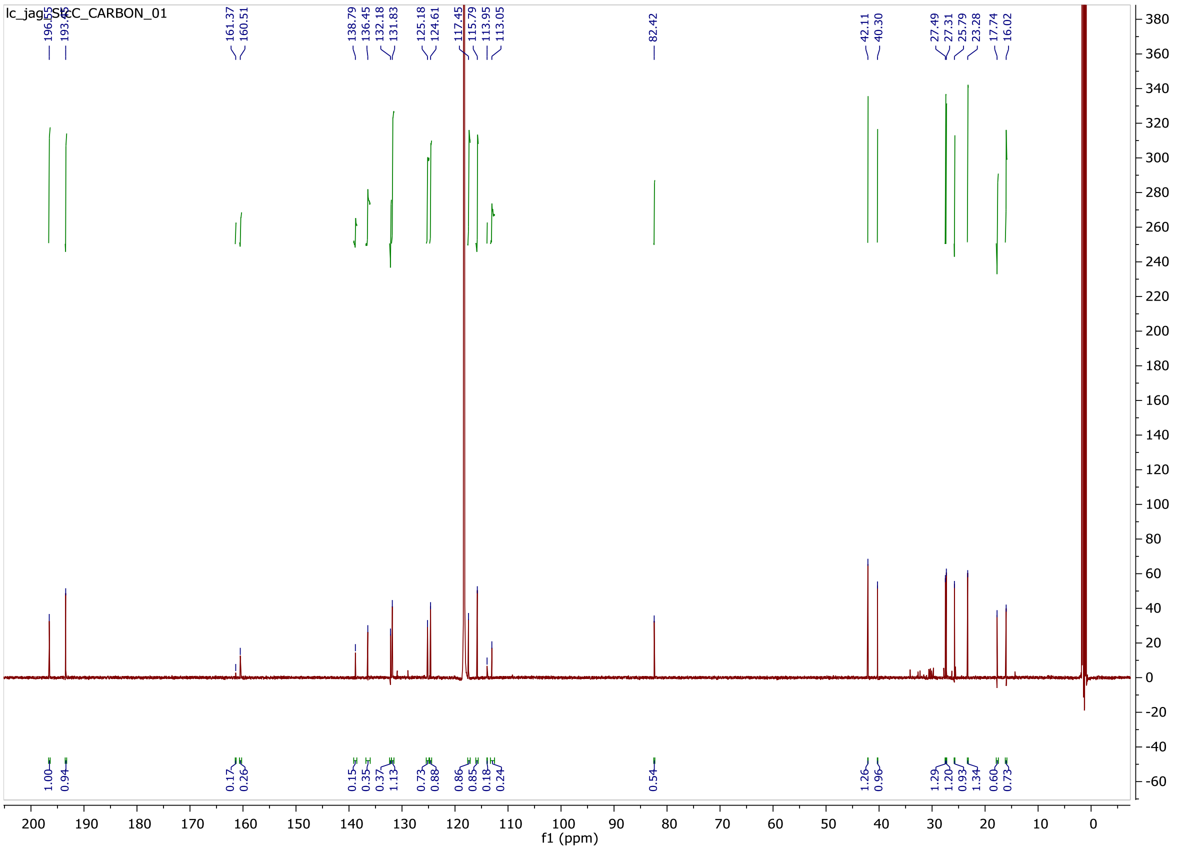

Fig. S14 ^{13}C (150 MHz) spectrum of **3** in CD_3CN.

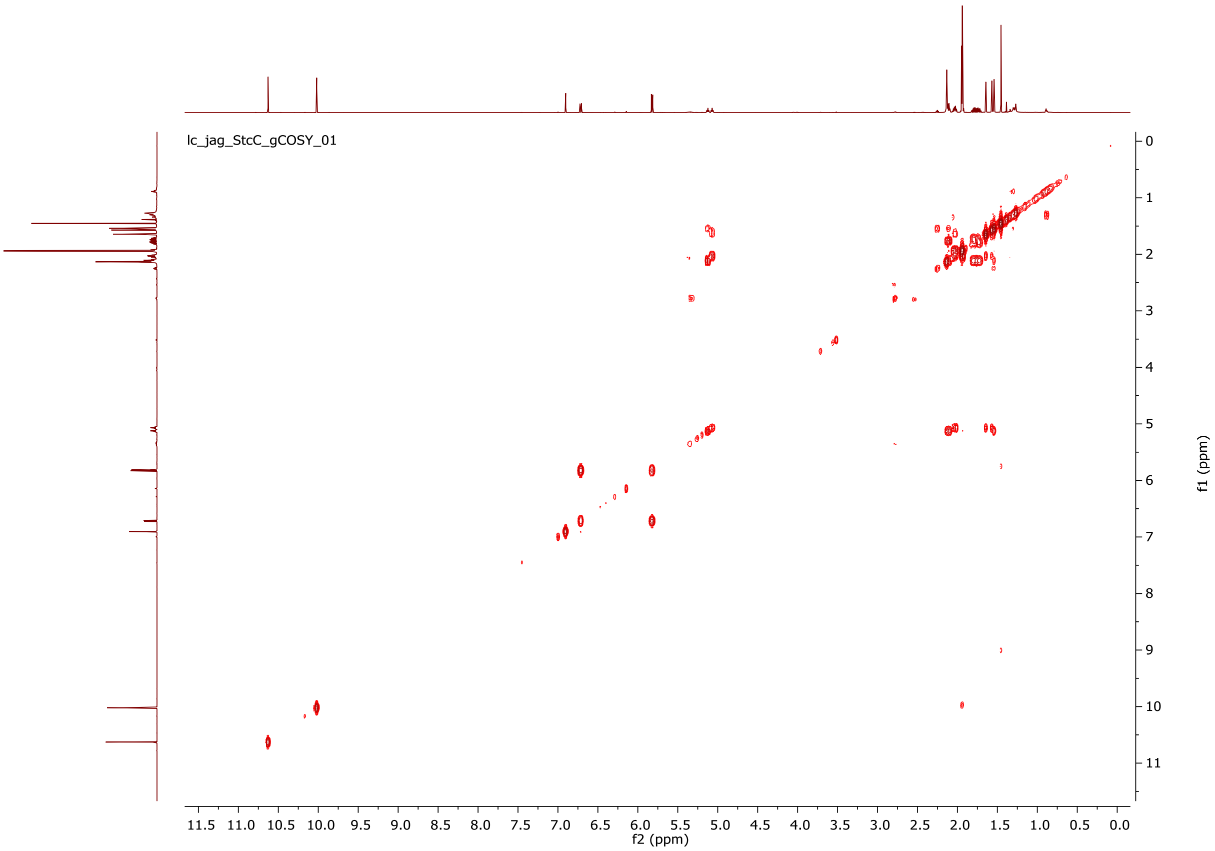

Fig. S15 ^{1}H-^{1}H COSY spectrum of **3** in CD_3CN.

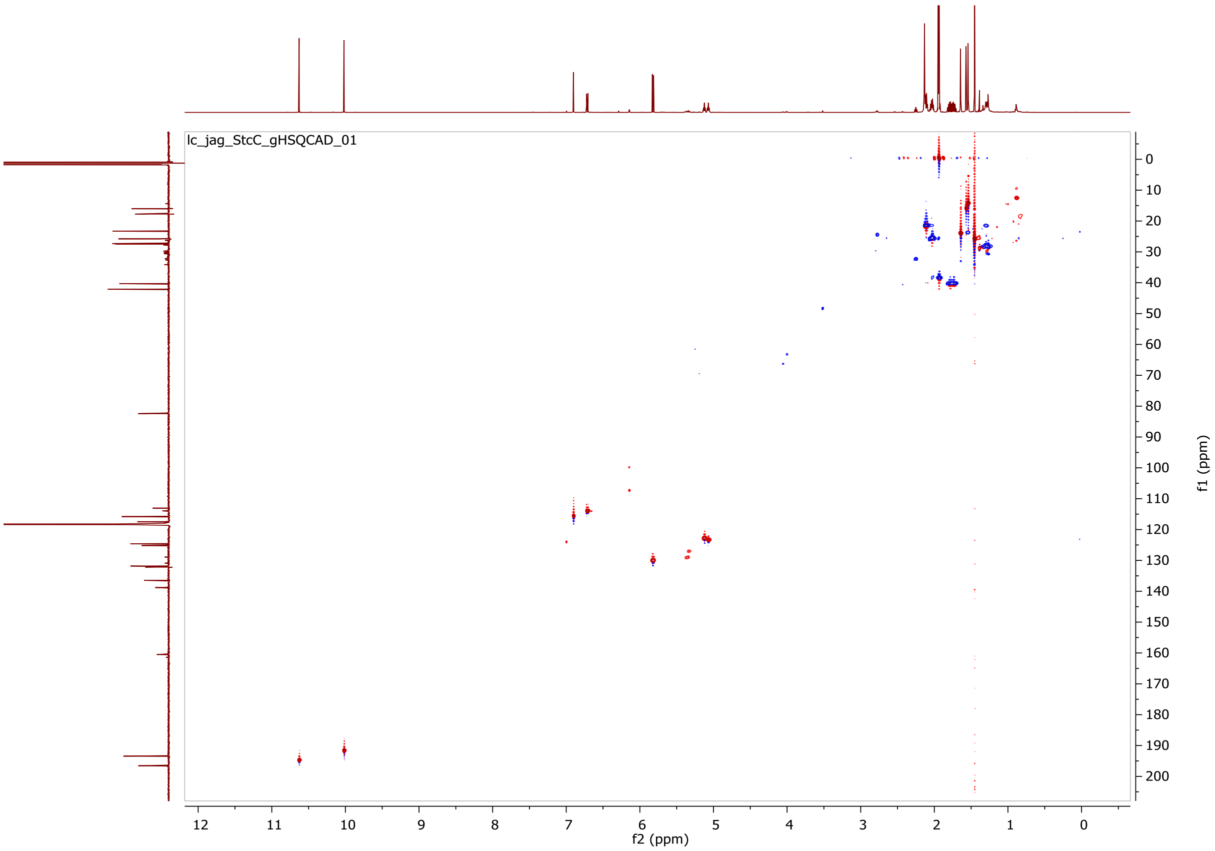

Fig. S16 HSQC spectrum of **3** in CD_3CN.

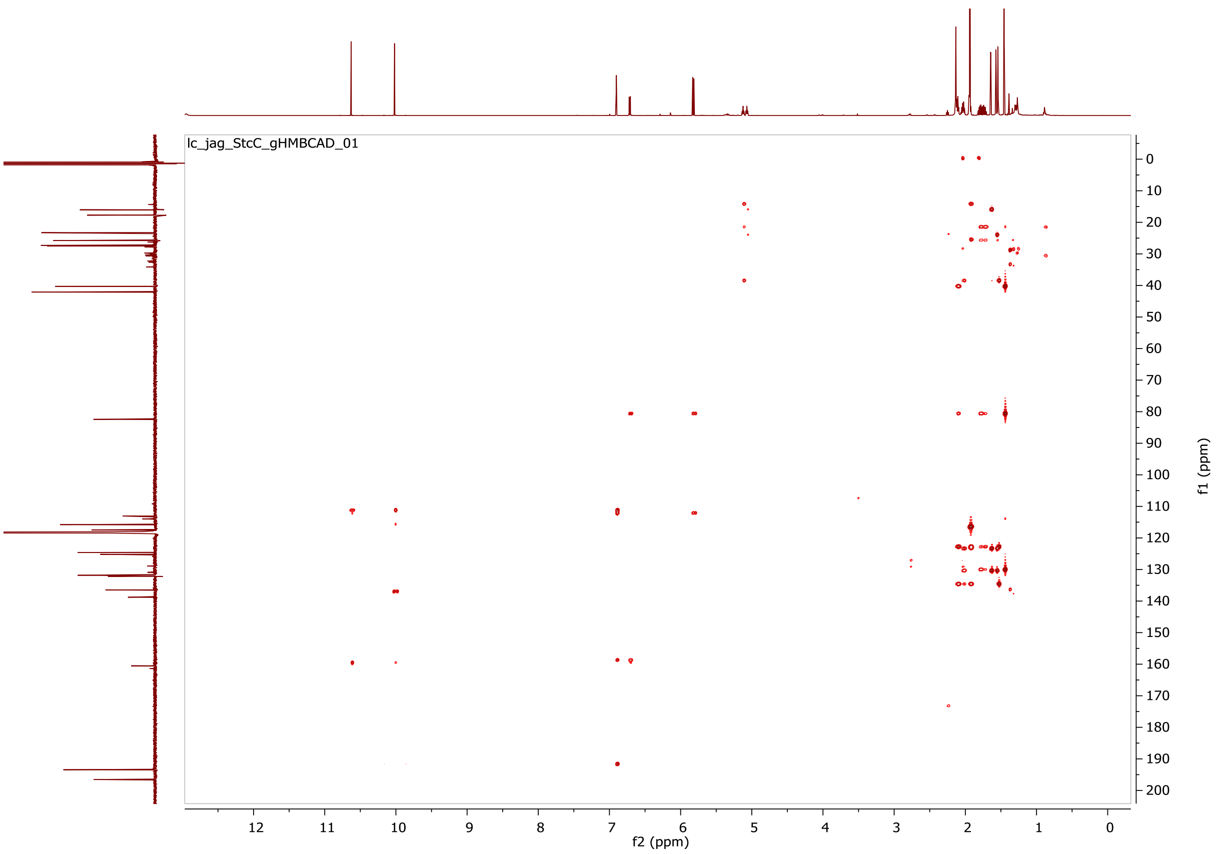

Fig. S17 HMBC spectrum of **3** in CD_3CN.

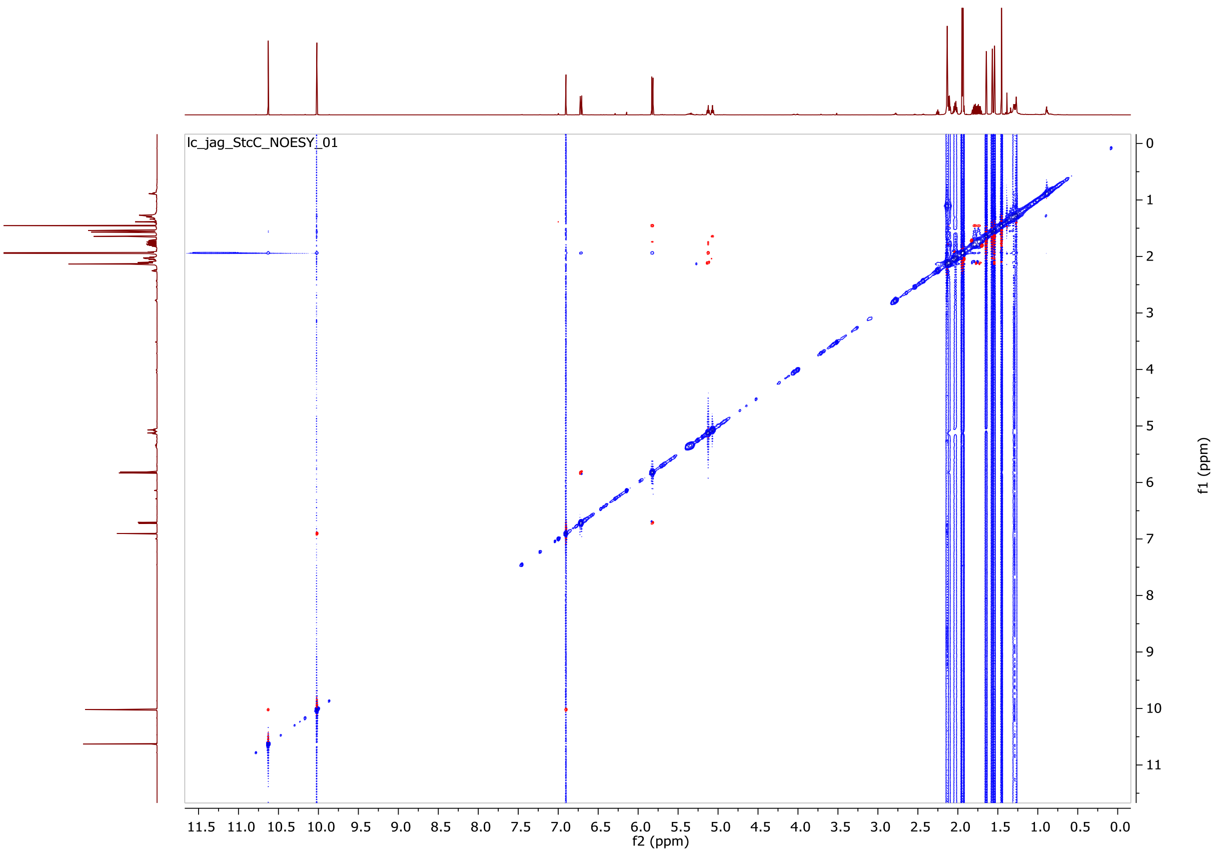

Fig. S18 2D NOESY spectrum of **3** in CD_3CN.

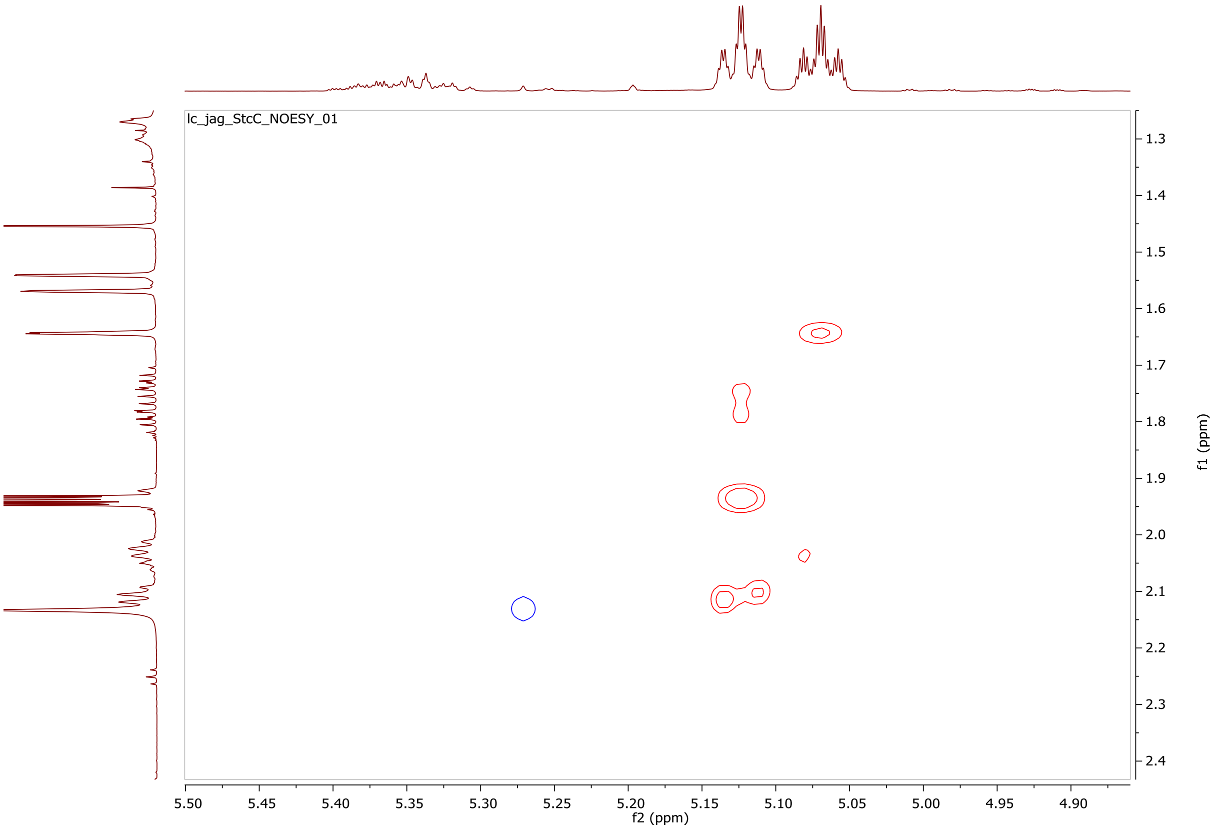

Fig. S19 2D NOESY section of **3** in CD_3CN.

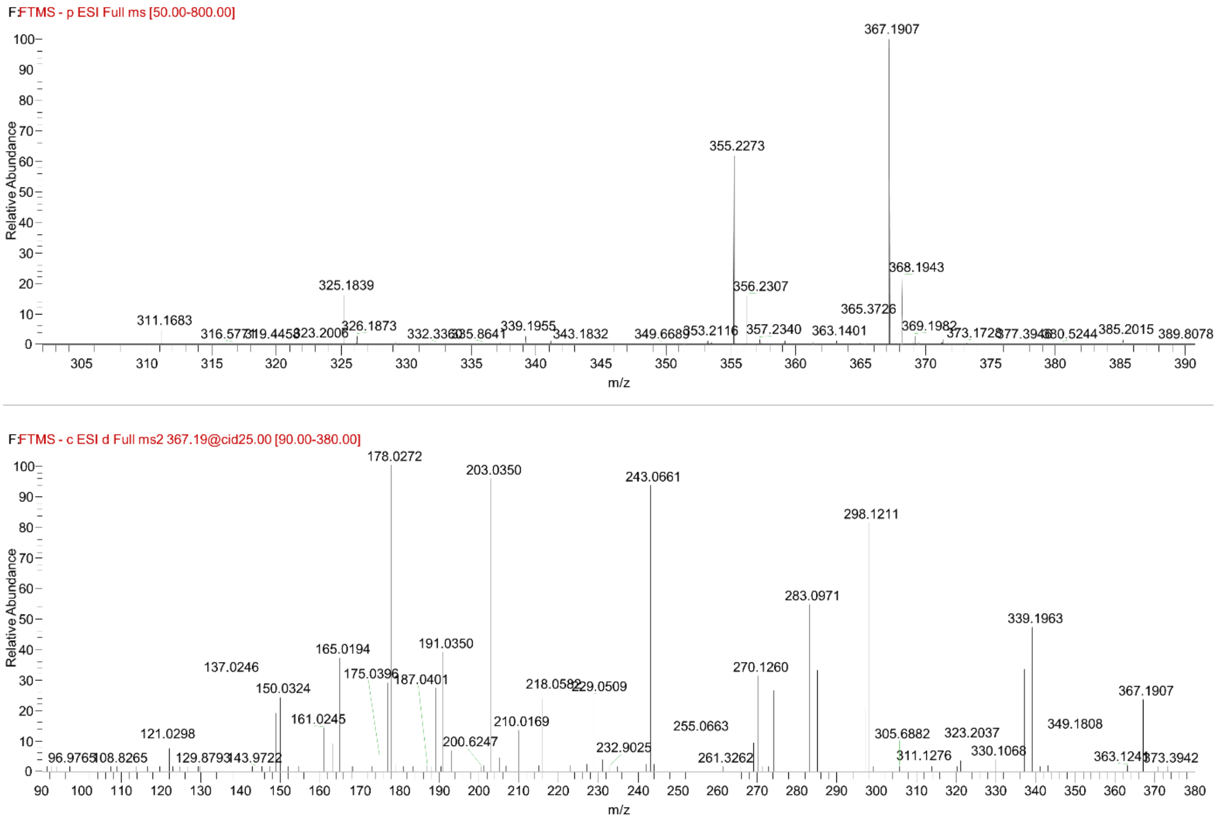

Fig. S20 HR-ESI-MS and fragmentation spectra of **3**.

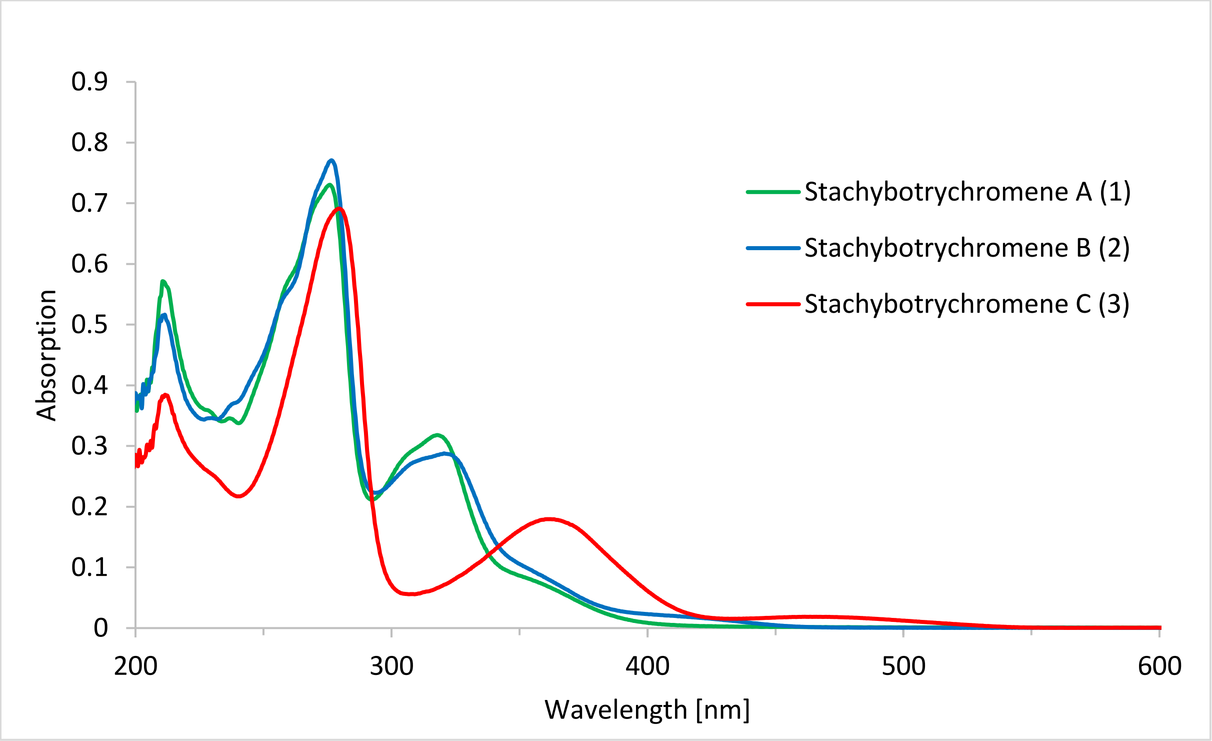

Fig. S21 UV-spectra of **1-3** in MeCN.

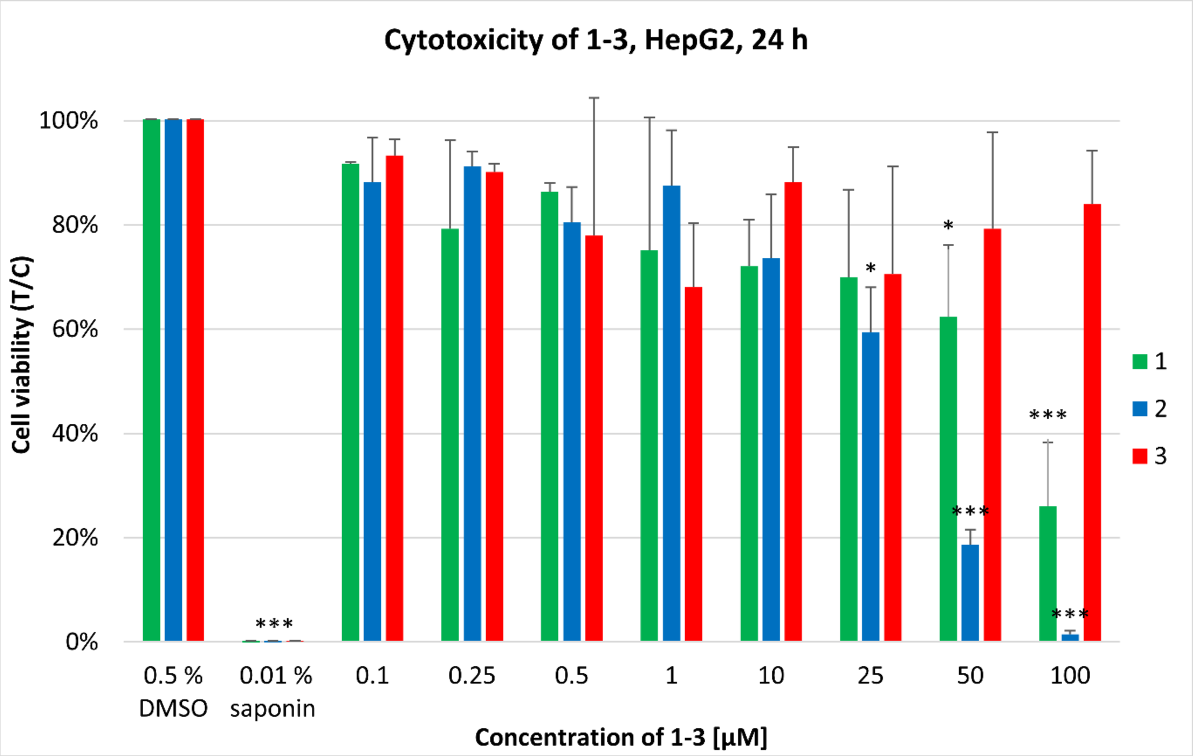

Fig. S22 Cell viability (T/C) in % of HepG2 cells after incubation of **1**-**3** for 24 h determined by resazurin reduction assay. Incubations were performed in sextuplicates from three independent passages (n=3); 0.5 % DMSO was used as negative control and 0.01 % saponin served as positive control. Statistically significance values * ($p \leq 0.05$), ** ($p \leq 0.01$), *** ($p \leq 0.001$) were compared to the lowest concentration.
